# The MarR Family Transcriptional Regulator EmrR Negatively Regulates the Type III Secretion System (T3SS) and Positively Modulates Pathogenicity in *Dickeya oryzae*

**DOI:** 10.1111/mpp.70255

**Published:** 2026-04-06

**Authors:** Mingfa Lv, Wenqi Huang, Ziwei Meng, Xiaojing Fan, Peng Li, Zeling Xu, Shaohua Chen, Xiaoyuan Chen, Jianuan Zhou, Lianhui Zhang, Yingying Cheng, Tao Zhuo, Ming Hu

**Affiliations:** ^1^ College of Plant Protection Fujian Agriculture and Forestry University Fuzhou Fujian China; ^2^ Ministry of Education Key Laboratory for Ecology of Tropical Islands, Hainan Provincial Key Laboratory for Tropical Plant and Animal Ecology, College of Life Sciences Hainan Normal University Haikou China; ^3^ Integrative Microbiology Research Centre, Guangdong Provincial Key Laboratory of Microbial Signals and Disease Control South China Agricultural University Guangzhou China; ^4^ Guangdong Provincial Key Laboratory of Utilization and Conservation of Food and Medicinal Resources in Northern Region Shaoguan University Shaoguan China; ^5^ Shenzhen Key Laboratory of Forensics BGI Forensic Shenzhen China

**Keywords:** biofilm, c‐di‐GMP, *Dickeya oryzae*, motility, pathogenicity, type III secretion system

## Abstract

The pathogen *Dickeya oryzae* is responsible for soft rot disease in both monocotyledonous and dicotyledonous plants, resulting in substantial yield losses across a variety of important crops. However, the molecular mechanisms and signalling pathways that regulate bacterial virulence remain poorly understood. In this study, we identified a conserved transcriptional regulator, EmrR (multidrug efflux transporter EmrAB transcriptional repressor), in *Dickeya* species that plays a critical role in controlling cellular motility. Phenotypic analysis demonstrated that EmrR is also involved in the regulation of cellulase, as well as biofilm formation. *emrR*deletion mutants displayed markedly decreased virulence in rice seed germination and potato tubes. Further investigation revealed that EmrR negatively regulates the expression of genes encoding the type III secretion system by decreasing the intracellular concentration of c‐di‐GMP (bis‐(3′‐5′)‐cyclic dimeric guanosine monophosphate) and also modulates the key regulatory gene *hrpL* within this system. DNase I footprinting confirmed that EmrR binds specifically to a sequence (5′‐CATTCGTTACTATGCAGGTTG‐3′) in the *emrR* promoter region. This finding highlights EmrR as a conserved and essential regulator of virulence in *Dickeya*, providing novel insights into the complex signalling and regulatory networks that regulate physiological processes, biochemical mechanisms and virulence development in this pathogen.

## Graphical abstract

This study revealed that the transcriptional regulator EmrR in *Dickeya oryzae* modulates virulence by regulating *hrpL* expression and c‐di‐GMP levels. It affects motility, biofilm formation, cellulase production and pathogenicity. EMSA and footprinting confirmed EmrR binds directly to the promoter of its target operon.

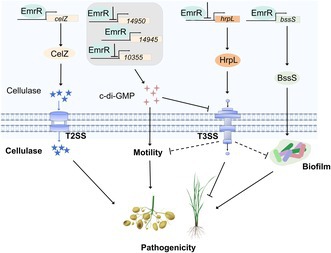

## 1 Introduction

The *Dickeya* genus is recognised as one of the 10 most significant bacterial pathogens globally. The soft rot disease it causes leads to substantial yields losses in both grain and economically important crops (Mansfield et al. 2012; Van der Wolf et al. 2014; Zhang et al. 2014; Li et al. 2020; Huang et al. 2021). Currently, the genus *Dickeya* comprises 12 species: *Dickeya chrysanthemi* , *D. dadantii* , *D. dianthicola* , *D. zeae* , *D*. *solani*, *D*. *fangzhongdai*, *D. aquatica* , *D*. *poaceaephila*, *D. lacustris* , *D*. *undicola*, *D. oryzae* and *D*. *parazeae* (Hu et al. 2018; Hugouvieux‐Cotte‐Pattat et al. 2019; Oulghazi et al. 2019; Wang et al. 2020; Hugouvieux‐Cotte‐Pattat and Van Gijsegem 2021). *D. oryzae* , previously known as *Erwinia chrysanthemi* pv. *zeae*, infects a wide range of monocotyledonous and dicotyledonous plants, causing soft rot in agricultural and ornamental species worldwide (Samson et al. 2005; Hussain et al. 2008; Wang et al. 2020). Extensive research on *D. dadantii* has elucidated the virulence and pathogenic mechanism of *Dickeya* species, revealing their ability to produce a variety of virulence factors. These mainly include plant cell wall‐degrading enzymes (PCWDEs) (Reverchon et al. 1994; Reverchon and Nasser 2013; Van der Wolf et al. 2014; Zhou et al. 2015), biofilm formation, exopolysaccharides (EPS), iron assimilation system (Enard et al. 1988), cell motility and chemotaxis (Antunez‐Lamas et al. 2009) and the antioxidant blue pigment indigotin (Enard et al. 1988; Reverchon et al. 2002).

The type III secretion system (T3SS) is highly conserved and encoded by the *dsp*/*hrp*/*hrc* gene clusters, which play a crucial role in the early stages of pathogen infection in the *Dickeya* genus. The expression of *hrp* genes is repressed in a nutrient‐rich medium, but is induced under nutrient‐deficient conditions and during plant infection (Wei et al. 1992). At least two predominant regulatory pathways have been characterised that are responsible for the activation of the T3SS in *Dickeya* (Yap et al. 2005; Tang et al. 2006; Yang et al. 2008; Li et al. 2009). HrpL is a central regulator within the HrpX/Y‐HrpS‐HrpL regulatory pathway and activates multiple downstream genes involved in the T3SS regulatory cascade, such as *hrpA*, *dspE* and *hrpN*, which encode a structural protein of the T3SS pilus, a T3SS effector and a T3SS harpin, respectively (Chatterjee et al. 2002; Tang et al. 2006). The two‐component system HrpX/HrpY activates the *hrpS* gene, which encodes a σ54‐enhancer binding protein. Upon activation, HrpS interacts with the RpoN‐containing RNA polymerase holoenzyme to initiate transcription of *hrpL* (Chatterjee et al. 2002; Yap et al. 2005; Tang et al. 2006). In the Rsm‐mediated regulatory pathway, the small RNA‐binding protein RsmA binds to *hrpL* mRNA, leading to its destabilisation (Zeng et al. 2010). In addition to the aforementioned pathway, the GacS‐GacA‐RsmB‐RsmA pathway exerts post‐transcriptional regulation on *rsmB* expression, resulting in the inactivation of RsmA and a subsequent increase in *hrpL* mRNA levels (Yang et al. 2008; Zeng et al. 2012; Yuan et al. 2020). Furthermore, SlyA acts as a parallel regulator of the *hrp* genes within the HrpL regulon in *D. dadantii* . As a member of the SlyA/MarR family transcriptional regulator, SlyA positively modulates the expression of *hrpA* and *hrpN* while negatively modulating *hrpL* through the downregulation of *hrpS* and upregulation of *rsmA* (Zou et al. 2012).

The regulatory mechanisms underlying the physiology and virulence of *D. oryzae* have been previously studied and are known to involve a complex and multilayered transcriptional regulatory network (Zhou et al. 2024). This network integrates multiple global regulators and signalling pathways to coordinate bacterial metabolism, stress adaptation and pathogenicity. These include the MarR family transcription factors such as SlyA and OhrR, and the Fis family transcription factor Fis (Zhou et al. 2016; Lv et al. 2018; Lv, Chen, et al. 2022; Lv, Ye, et al. 2022). Additionally, several two‐component regulatory systems (TCSs) have been identified, including the quorum‐sensing (QS) signal responder VfmIH, the GacAS type system TzpSA and the ArcBA system involved in anoxic redox regulation (Lv et al. 2019; Chen et al. 2022; Lv, Chen, et al. 2022; Lv, Ye, et al. 2022). Other regulatory systems implicated in the regulation of *D. oryzae* include the acyl‐homoserine lactone (AHL)‐mediated QS system (Hussain et al. 2008), polyamines‐mediated host–pathogen communication (Shi et al. 2019), c‐di‐GMP signalling pathway (Chen et al. 2016, 2020), the conserved RNA chaperone protein Hfq (Shi et al. 2022) and the Lrp/AsnC family transcriptional regulator Lrp (Wu et al. 2025). Together, these studies highlight the complexity of the transcriptional regulatory network in *D. oryzae* and suggest that additional, yet uncharacterised regulators may play important roles in coordinating virulence and pathogenicity.

Genome sequence analysis has revealed that *D. oryzae* EC1 possesses at least 185 transcription factors (Zhou et al. 2015), many of which remain functionally uncharacterised. In this study, we focused on previously uncharacterised MarR family transcriptional regulators. We discovered that the swimming motility of a deletion mutant was significantly decreased and identified it as EmrR. Given the established roles of MarR (multiple antibiotic resistance regulator) family regulators in controlling multidrug resistance, stress responses and virulence‐related traits in diverse bacterial pathogens (Deochand and Grove 2017), we hypothesised that EmrR may play an important role in regulating virulence‐associated phenotypes in *D. oryzae* .

To test this hypothesis, we investigated the potential regulatory roles of EmrR in bacterial motility, expression of the multidrug efflux pump genes *emrAB*, production of cellulase, biofilm formation, intracellular c‐di‐GMP levels and the transcription of T3SS genes. Through these analyses, this study aimed to elucidate the contribution of EmrR to the transcriptional regulatory network governing physiology and pathogenicity in *D. oryzae* EC1.

## 2 Results

### 2.1 Deletion of *emrR* Decreases Swimming Motility and Swarming Motility

**FIGURE 1:**
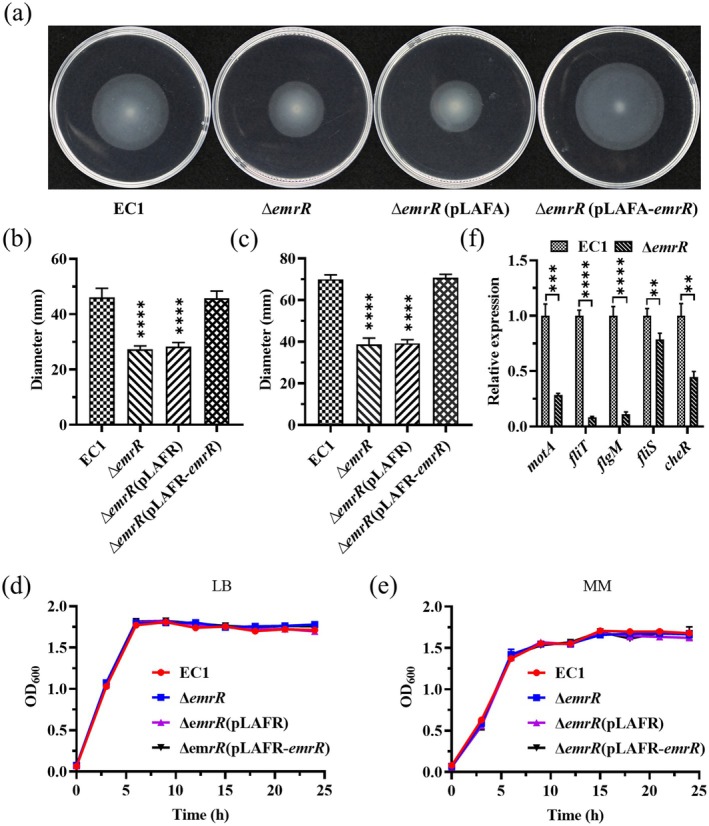
The *emrR* deletion mutant decreased the cell motility of *Dickeya oryzae* EC1. (a) Swimming motility assay of wild‐type strain EC1 and its derivative strains. Δ*emrR*(pLAFR‐*emrR*), complemented strain with wild‐type *emrR* gene in pLAFR3. (b) Statistical analysis of swimming motility diameter. (c) Statistical analysis of swarming motility diameter. (d) Determination of growth kinetics in Luria Bertani medium. (e) Determination of growth kinetics in minimal medium. (f) The expression levels of *motA*, *fliT*, *flgM*, *fliS* and *cheR*, which encode the flagellar motor stator protein MotA, the flagellar biosynthesis regulatory protein FliT, the anti‐sigma factor FlgM, the flagellar export chaperone FliS and the chemotaxis protein CheR, respectively. Experiments were repeated at least three times in triplicate and errors indicate standard deviation. **p < 0.01, ***p < 0.001, ****p < 0.0001, Student's *t* test.

To investigate the roles of the uncharacterised MarR family transcriptional regulatory genes in *D. oryzae* EC1, deletion mutants annotated as MarR family transcriptional regulatory proteins were created, and the swimming motility was assayed. We observed that a deletion mutant of the gene *W909_RS15260* exhibited significantly reduced swimming and swarming motility compared to the wild‐type strain EC1. The *W909_RS15260* gene encodes a protein consisting of 170 amino acids (RefSeq id WP_016941676.1), which shows high sequence similarity (100% query coverage and 75.9% identity) to the EmrR transcriptional repressor of the multidrug efflux transporter EmrAB in *Escherichia coli* (RefSeq id WP_074014164.1). When the pLAFR3 plasmid carrying the wild‐type strain EC1 *W909_RS15260* gene was introduced into the deletion mutants of *W909_RS15260*, both swimming and swarming motility were restored to the level comparable to those observed in the wild‐type strain EC1 and EC1 (pLAFR3) strains (Figure 1a–c).

In *E. coli* , EmrR serves as a transcriptional repressor located upstream of the multidrug resistance pump genes *emrAB*, which protects the cell from several unrelated antimicrobial chemicals and binds to the operon of *emrRAB* to modulate negatively their transcription (Lomovskaya et al. 1995; Xiong et al. 2000). In *D. oryzae* EC1, we found that *W909_RS15260* is a locus upstream of *W909_RS15265* and *W909_RS15270*, which are annotated as multidrug export protein EmrA and multidrug efflux MFS transporter subunit EmrB, respectively. Therefore, the *D. oryzae* gene *W909_RS15260* was designated as *emrR* as well. The mutant strain ∆*emrR* exhibited comparable growth patterns to the wild‐type strain EC1 in Luria Bertani (LB) and minimal medium (MM) (Figure 1d,e). We also conducted reverse transcription‐quantitative PCR (RT‐qPCR) to assess the expression levels of *motA*, *fliT*, *flgM*, *fliS* and *cheR*, which encode the flagellar motor stator protein MotA, the flagellar biosynthesis regulatory protein FliT, the anti‐sigma factor FlgM, the flagellar export chaperone FliS and the chemotaxis protein CheR, respectively. The results demonstrated a significant decrease in their expression in ∆*emrR* compared to the wild‐type strain EC1 (Figure 1f), thereby confirming the pivotal role of EmrR in positively regulating bacterial motility and prompting further investigation into the regulatory role of *emrR* in other functions.

### 2.2 EmrR Negatively Regulates *emrRAB* Transcription in *D. oryzae*

EmrR, a member of the MarR family of transcriptional repressors, represses the transcription of the *emrAB* operon by directly binding to its promoter in *E. coli* (Lomovskaya et al. 1995; Xiong et al. 2000). Genome analysis of 12 species showed that the EmrR‐EmrAB system is prevalent in *Dickeya* (Figure S1). To further investigate the phylogenetic relationships of EmrR among different *Dickeya* species, we performed a phylogenetic analysis based on the amino acid sequence of EmrR from 12 representative *Dickeya* species. The resulting EmrR‐based phylogeny was then compared with a genome‐based phylogeny constructed using conserved single‐copy orthologous genes. The results revealed that the overall clustering patterns were highly consistent between the two phylogenetic trees. Notably, *D. oryzae* EC1 grouped closely with *D. parazeae* Ech586 in both analyses. These results indicate that EmrR is highly conserved within the genus *Dickeya*, and that EmrR from *D. oryzae* EC1 is most closely related to that from *D. parazeae* Ech586 (Figures S1 and S2).

**FIGURE 2:**
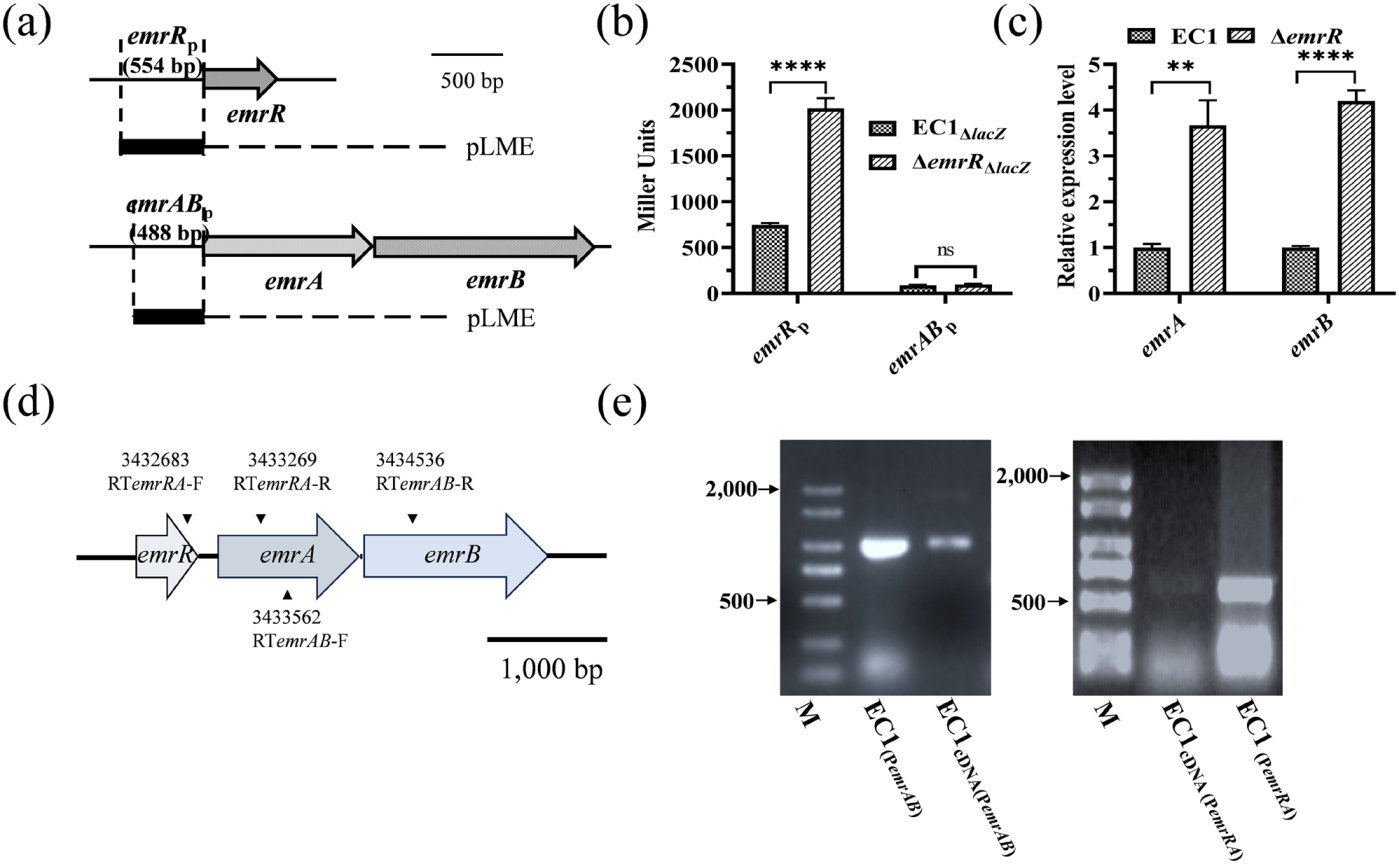
*emrR* and *emrAB* form an operon. (a) Schematic representation of the cloned *emr*‐*lacZ* fusions. The promoter regions of *emrR* and *emrAB* were cloned into the vector pLME. (b) β‐galactosidase activity. (c) The expression levels of genes *emrA* and *emrB*. (d) Schematic illustration of the reverse transcription (RT)‐PCR primers design. (e) RT‐PCR assay. Experiments were conducted in triplicate and repeated at least three times; the errors indicate standard deviation. **p < 0.01, ****p < 0.0001, Student's *t* test.

To elucidate the regulatory mechanism of EmrR on both the *emrAB* operon and itself, a reporter assay was conducted by fusing a 554‐bp promoter region, containing a 30‐bp coding region of *emrR* and a 488‐bp promoter region, containing a 51‐bp coding region of *emrAB*, upstream of the promoterless *lacZ* gene in plasmid pLME (Figure 2a). The reporter plasmids pLME‐*emrR*_p_ and pLME‐*emrAB*_p_ were then transferred into wild‐type strain EC1_∆*lacZ*_ and *emrR* mutant strain ∆*emrR*_∆*lacZ*_, followed by measurement of β‐galactosidase activity. The results showed that ∆*emrR*_∆*lacZ*_ (pLME‐*emrR*_p_) exhibited significantly higher levels of β‐galactosidase activity compared to EC1_∆*lacZ*_(pLME‐*emrR*_p_); however, there was no significant difference between ∆*emrR*_∆*lacZ*_ (pLME‐*emrAB*_p_) and EC1_∆*lacZ*_ (pLME‐*emrAB*_p_) (Figure 2b). RT‐qPCR analysis was conducted to evaluate the expression levels of *emrA* and *emrB* in the *emrR* mutant. The results indicated that their expression increased approximately 2.5‐ and 3‐fold, respectively, compared to the wild‐type strain EC1 (Figure 2c). Additionally, using mRNA as the template, RT‐PCR amplification was carried out with primers *emRA*‐F/R and *emrAB*‐F/R. The results demonstrated that the target product fragments were successfully amplified in both cases (Figure 2d,e). These findings indicate that the *emrR*‐*emrAB* gene cluster constitutes an operon and shares a common promoter.

### 2.3 Deletion of *emrR* Alters the Activity of Cellulase

**FIGURE 3:**
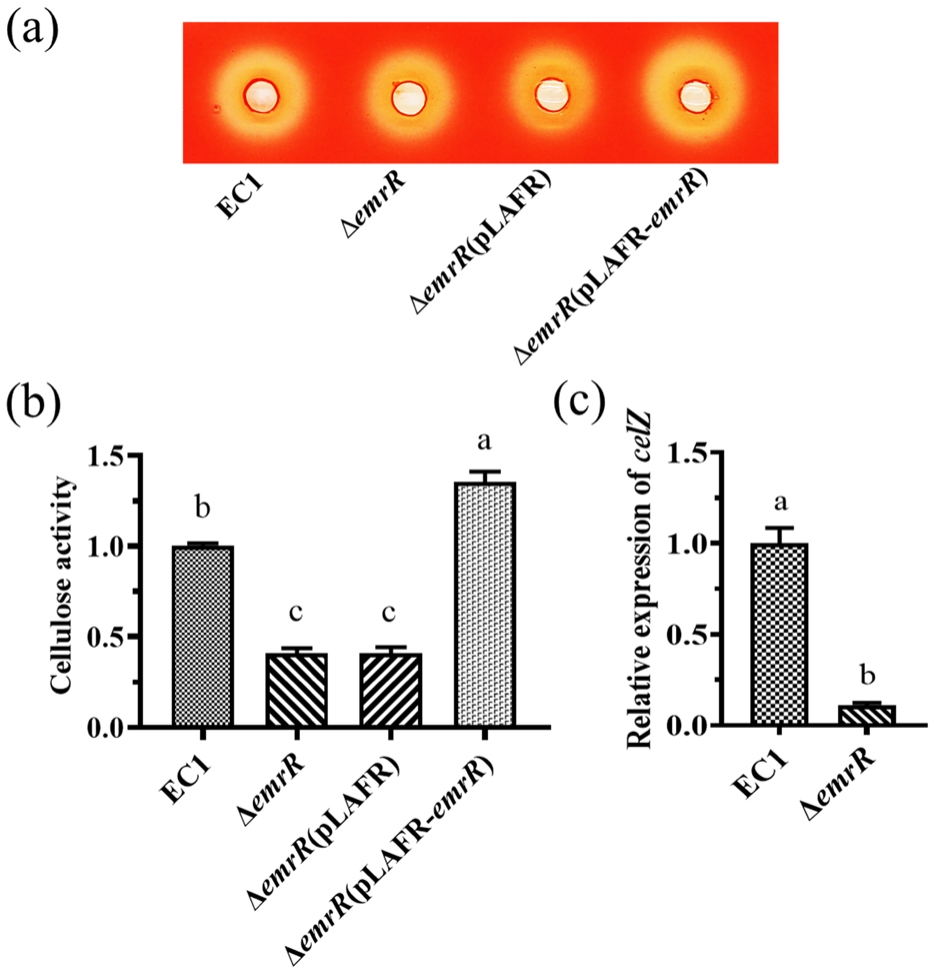
EmrR modulates the production of cellulase. (a) Qualitative detection of cellulase activity in the assay plate. (b) Quantitative determination of extracellular cellulase activity in wild‐type strain EC1 and its derivative strains. The results of the mutant ∆*emrR* and its complemented strain ∆*emrR*(pLAFR‐*emrR*) were normalised relative to those of the wild‐type EC1, which was designated as 1 for comparative purposes. (c) The expression level of gene *celZ*. The experiments were conducted in triplicate and repeated at least three times, with error bars representing the standard deviation. Statistical analysis was carried out for each data group, and values that are significantly different (ANOVA, *p* < 0.05) are denoted by different letters.

In the *Dickeya* genus, the PCWDEs (e.g., cellulases, pectinases and proteinases) play a crucial role in producing soft rot symptoms by damaging the cellular structure of the host (Hugouvieux‐Cotte‐Pattat et al. 1996; Ma et al. 2007). Additionally, zeamine functions as a key virulence factor by inhibiting rice seed germination and seeding growth (Zhou et al. 2011; Lv et al. 2019). To determine how the *emrR* gene affects extracellular enzyme production, we employed both qualitative and quantitative methods. Results showed that the *emrR* deletion mutant had significantly altered production of cellulase, which was reduced by about 4‐fold (Figure 3a,b) compared with that of the wild‐type strain EC1 and the complemented mutant strain carrying the wild‐type *emrR* gene (Figure 3a,b). However, the activity of pectinases and proteinases in the *emrR* mutant did not show marked alteration compared to the wild‐type strain EC1 (Figure S3). Similarly, the production of zeamine was not significantly affected in ∆*emrR* compared to the wild‐type strain EC1 and the complemented strain ∆*emrR*(pLAFR‐*emrR*) (Figure S4a). To further investigate the expression of key genes, we conducted RT‐qPCR analysis. The results showed that the expression level of *celZ* was decreased 4‐fold in the *emrR* mutant compared to wild‐type strain EC1, but the expression levels of *zmsA* and *zmsK* were comparable to those observed in the wild‐type strain EC1 in the mutant ∆*emrR* (Figure 3c and Figure S4b).

### 2.4 Deletion of *emrR* Reduces Biofilm Formation

**FIGURE 4:**
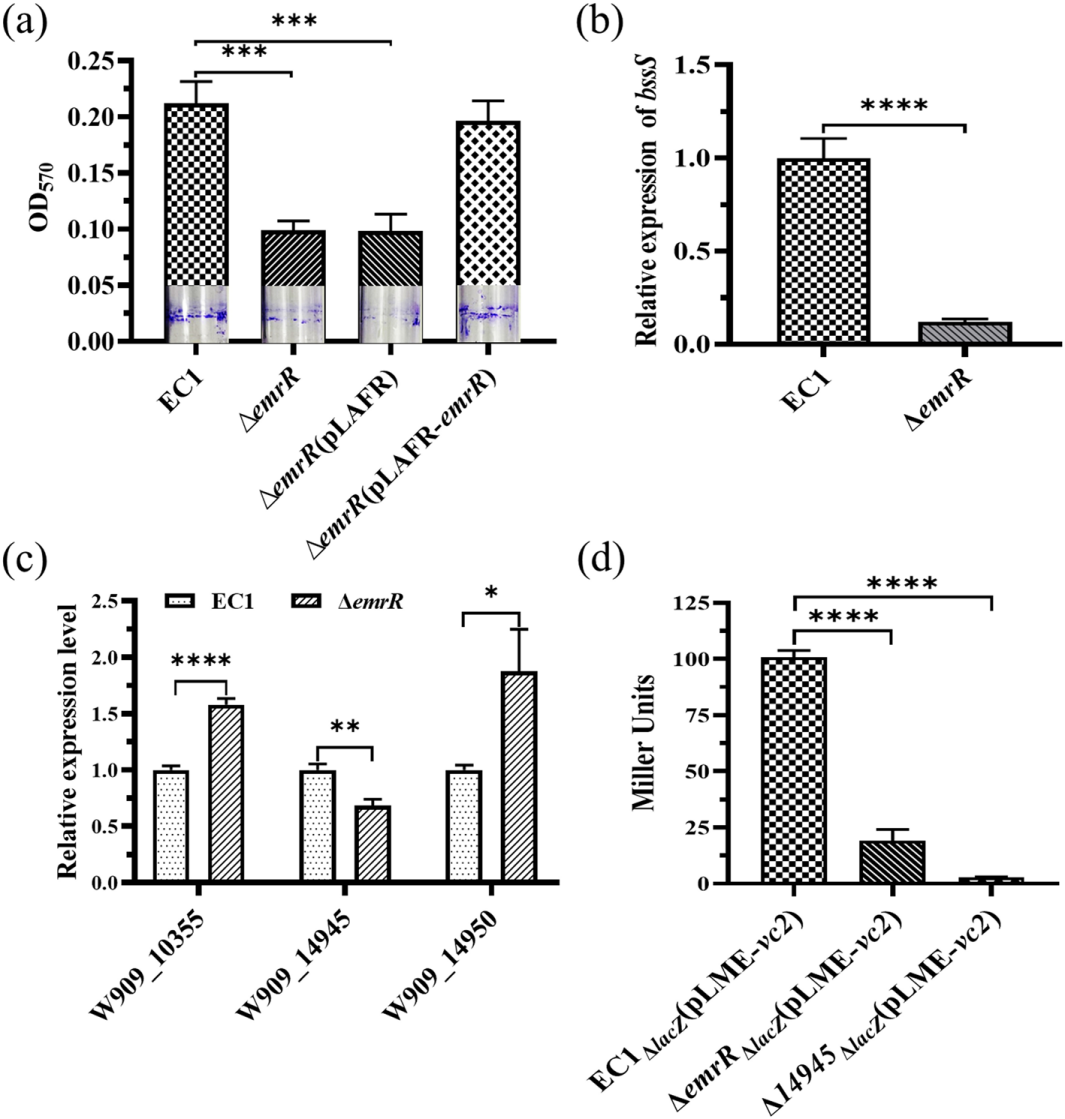
EmrR modulates biofilm formation and the intracellular concentration of c‐di‐GMP. (a) Qualitative and quantitative determination of the biofilm formation. (b) The expression level of *bssS,* encoding a regulatory protein involved in modulating biofilm formation. (c) Determination of expression levels of key c‐di‐GMP biosynthetic gene *W909_14945* and degradation genes *W909_10355* and *W909_14950*. (d) Determination of intracellular c‐di‐GMP concentration in wild‐type strain EC1, ∆*emrR* and ∆*14945* by the β‐galactosidase activity assay. All experiments were performed in triplicate and independently repeated at least three times, with the errors reported representing the standard deviation. *p < 0.1, **p < 0.01, ***p < 0.001, ****p < 0.0001, Student's *t* test.

Biofilm formation is an environmental adaptation and is recognised as a mode of growth that increases bacterial resistance to antimicrobial agents and makes bacterial cells less susceptible to host defence mechanisms, and so enables pathogens to survive in hostile environments, as well as disperse and colonise new niches (Del Pozo 2018). To determine the role of EmrR in biofilm formation, we quantified adhered biofilm biomass using crystal violet staining. The deletion mutant of *emrR* demonstrated about 2.2‐fold lower capacity to form biofilm than the wild‐type strain EC1 and the complemented strain ∆*emrR*(pLAFR‐*emrR*) (Figure 4a). The expression of *bssS*, which encodes a regulatory protein involved in modulating biofilm formation, was assessed using RT‐qPCR. The results revealed an 8‐fold decrease in *bssS* expression in the *emrR* mutant compared to the wild‐type strain EC1 (Figure 4b).

Previous studies showed that the ubiquitous second messenger c‐di‐GMP is implicated in modulating biofilm formation and cellular motility in numerous bacterial pathogens. In *D. oryzae* , the gene *W909_14945* is implicated in the synthesis of c‐di‐GMP, whereas *W909_10355* and *W909_14950* are implicated in its degradation (Chen et al. 2022). The gene expression of *W909_14945*, *W909_10355* and *W909_14950* was examined using RT‐qPCR. The results demonstrated that the synthesis‐related gene *W909_14945* decreased approximately 1.47‐fold, while the degradation‐associated genes *W909_10355* and *W909_14950* increased approximately 1.58‐ and 1.88‐fold, respectively, compared to wild‐type strain EC1 (Figure 4c). Subsequently, the reporter plasmid pLME‐*vc2* was constructed by fusing the c‐di‐GMP riboswitch *vc2* with the plasmid pLME and introduced into wild‐type strain EC1_∆*lacZ*_, ∆*emrR*_∆*lacZ*_, ∆*W909_14945*_∆*lacZ*_ to evaluate the intracellular concentration of c‐di‐GMP by β‐galactosidase activity. The results demonstrated a significant decrease in ∆*W909_14945*_∆*lacZ*_ and ∆*emrR*_∆*lacZ*_ strains, comparable levels to the wild‐type strain EC1_∆*lacZ*_ (Figure 4d). These results suggest that the change in biofilm formation capacity may also be attributed to alterations in the intracellular c‐di‐GMP homeostasis of the ∆*emrR* mutant.

### 2.5 Deletion of *emrR* Reduces the Virulence to Potato and Rice

**FIGURE 5:**
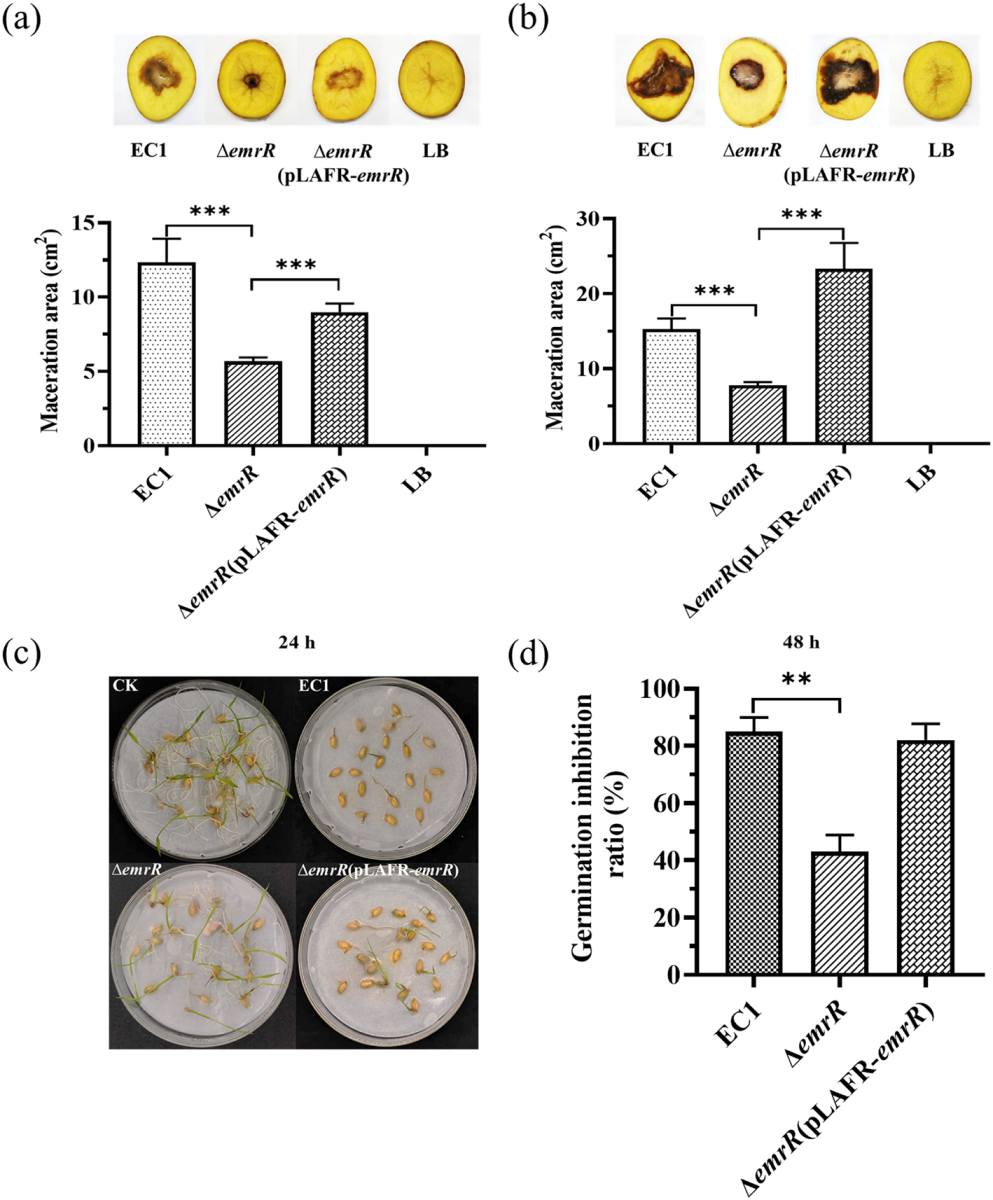
The *emrR* mutant exhibits reduced virulence on potato and rice seeds. (a) The maceration ability was assayed by measuring the maceration area on potato slices after 24 h of incubation and (b) after 48 h of incubation. The potato slice experiments were repeated at least three times in five, and errors indicate standard deviation. EC1, wild type; Δ*emrR*, deletion mutant; Δ*emrR*(pLAFR‐*emrR*), complemented strain; LB, Luria Bertani medium. (c) Rice seed germination treated with phosphate‐buffered saline (PBS), wild‐type strain EC1 and its derivative strains. CK, PBS control. The experiments were conducted in triplicate and repeated at least three times. (d) Statistics on the germination inhibition rate of wild‐type strain EC1 and its derivative strains. **p < 0.01, ***p < 0.001, Student's *t* test.

*Dickeya oryzae* EC1 causes infections in dicotyledonous plants and induces soft rot symptoms on potato tubers (Hussain et al. 2008). In order to evaluate the ability of *emrR* mutants to cause maceration and soft rot symptoms on potato tubers, cultures of 1 μL of OD_600_ = 1.0 derived from the wild‐type strain EC1, mutant strain ∆*emrR* and complemented strain ∆*emrR* (pLAFR‐*emrR*) were inoculated at the centre of potato tubers. The maceration area was observed and measured after 24 h and 48 h at 28°C. The maceration area caused by ∆*emrR* was significantly smaller compared to the wild‐type strain EC1, with reductions of approximately 4.5‐fold at 24 h and 3.7‐fold at 48 h. Moreover, complementation with ∆*emrR*(pLAFR‐*emrR*) restored the maceration area to approximately 73.2% and 152.3% of that induced by the wild‐type strain EC1 at 24 and 48 h (Figure 5a,b). Additionally, overexpression of *emrR* in the wild‐type strain EC1 resulted in an enhanced maceration area on potato slices compared to the wild‐type strain EC1 (Figure S5).

Efficient inhibition of rice germination and seedling growth by *D. oryzae* represents a crucial virulence trait. To evaluate the impact of *emrR* deletion on virulence to rice, rice seeds were inoculated with the wild‐type strain EC1, the mutant strain ∆*emrR* and the complemented strain ∆*emrR*(pLAFR‐*emrR*) at varying cell densities; each treatment involved 20 rice seeds, and phosphate‐buffered saline (PBS) was used as the control. The germination rate was assessed 1 week after incubation at 28°C under a photoperiod of 16 h of light and 8 h of darkness. The mutant strain ∆*emrR* exhibited a significantly weaker inhibitory effect on rice seed germination compared to the wild‐type strain EC1 and the complemented strain ∆*emrR*(pLAFR‐*emrR*), with almost no germination observed at a bacterial cell density of 10^3^ cfu (Figure 5c). In contrast, high germination was observed in rice seeds treated with sterile water (Figure 5c). Statistical analysis revealed that the germination inhibition rate was reduced to 43% compared to the wild‐type strain EC1 (Figure 5d). This finding suggests that EmrR plays a key regulatory role in the modulating of virulence in rice seeds.

### 2.6 EmrR Negatively Regulates Gene Expression of the Type III Secretion System

**FIGURE 6:**
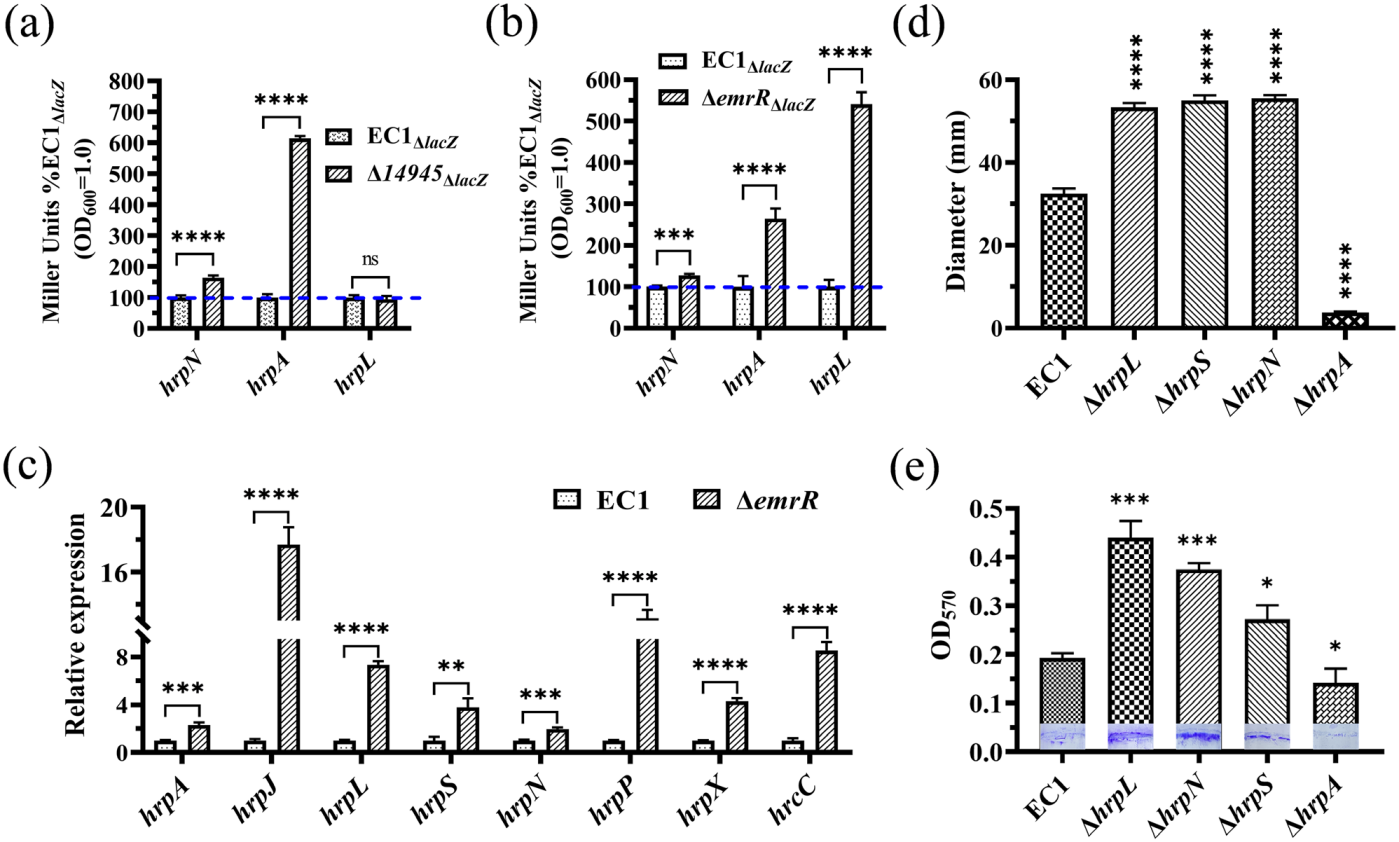
EmrR regulates the expression of the type III secretion system (T3SS) genes. (a) The expression levels of genes *hrpA*, *hrpN* and *hrpL* in the mutant ∆*14945* were analysed using the β‐galactosidase activity assay. (b) The expression levels of genes *hrpA*, *hrpN* and *hrpL* in the mutant ∆*emrR* were analysed using the β‐galactosidase activity assay. (c) The expression levels of T3SS genes *hrpA*, *hrpJ*, *hrpL*, *hrpS*, *hrpN*, *hrpP*, *hrpX* and *hrpC*, were analysed by reverse transcription‐quantitative PCR. (d) Statistical analysis of swimming motility in mutants ∆*hrpL*, ∆*hrpN*, ∆*hrpS* and ∆*hrpA*. (e) Evaluation of the biofilm formation by mutants ∆*hrpL*, ∆*hrpN*, ∆*hrpS* and ∆*hrpA*. All experiments were conducted in triplicate and independently repeated at least three times, with the reported errors representing the standard deviation. *p < 0.1, **p < 0.01, ***p < 0.001, ****p < 0.0001, Student's *t* test.

There is a significant correlation between the regulation of the T3SS and c‐di‐GMP signal transduction, with c‐di‐GMP signalling playing an essential role in modulating the expression of T3SS‐related genes (Yi et al. 2010; Yuan et al. 2018; Jiang et al. 2022). Therefore, the promoter region was fused to construct reporter plasmids for *hrpN*, *hrpA* and *hrpL*. These constructs were subsequently transformed into both the wild‐type strain EC1_∆*lacZ*_ and the key synthetic gene mutant ∆*W909_14945*_∆*lacZ*_ of c‐di‐GMP, and β‐galactosidase activity was measured at OD_600_ = 1.0 in MM. The expression levels of *hrpN*, *hrpA* and *hrpL* in the mutant ∆*W909_14945*, as determined by RT‐qPCR, were consistent with the β‐galactosidase activity results (Figure S6). Notably, the expression of *hrpN* and *hrpA* was significantly upregulated in the mutant, whereas *hrpL* expression showed no significant difference compared to the wild‐type strain EC1_∆*lacZ*_ (Figure 6a). These findings confirm that the intracellular concentration of c‐di‐GMP negatively regulates the expression of T3SS‐related genes in *D. oryzae* . Similarly, in the strain ∆*emrR*_∆*lacZ*_, the results of β‐galactosidase activity indicated that the expression levels of *hrpA*, *hrpN* and *hrpL* were significantly upregulated compared to that of the wild‐type strain EC1_∆*lacZ*_ (Figure 6b). Subsequently, the expression levels of several genes associated with the T3SS were quantitatively analysed using RT‐qPCR. The results demonstrated that in the ∆*emrR* mutant, the expression of *hrpA*, *hrpJ*, *hrpL*, *hrpS*, *hrpN*, *hrpP*, *hrpX* and *hrcC* were increased to 1.3‐, 16.7‐, 6.3‐, 2.8‐, 1.0‐, 12.1‐, 3.3‐, 7.5‐fold, respectively, compared to those in the wild‐type strain EC1 (Figure 6c). These results provide compelling evidence for the differential expression of genes encoding the T3SS observed in the *emrR* mutant.

Given the markedly increased expression levels of T3SS‐related genes and the substantially diminished motility observed in mutants ∆*emrR*, we generated deletion mutants for *hrpA*, *hrpN*, *hrpS* and *hrpL*. Subsequently, we evaluated their swimming motility, biofilm formation ability, and capacity to induce maceration on potato slices. The results demonstrated that both swimming motility and biofilm formation were markedly increased in mutants ∆*hrpN*, ∆*hrpS* and ∆*hrpL*, whereas they were significantly decreased in the mutant ∆*hrpA* (Figure 6d,e and Figure S7a). Similarly, the capacity to induce maceration on potato was significantly increased in mutants ∆*hrpN*, ∆*hrpS* and ∆*hrpL*, while no significant difference was observed in the ∆*hrpA* compared to the wild‐type strain EC1 (Figure S7b,c).

### 2.7 EmrR Directly Binds to the Promoter Region of Key Virulence Genes

**FIGURE 7:**
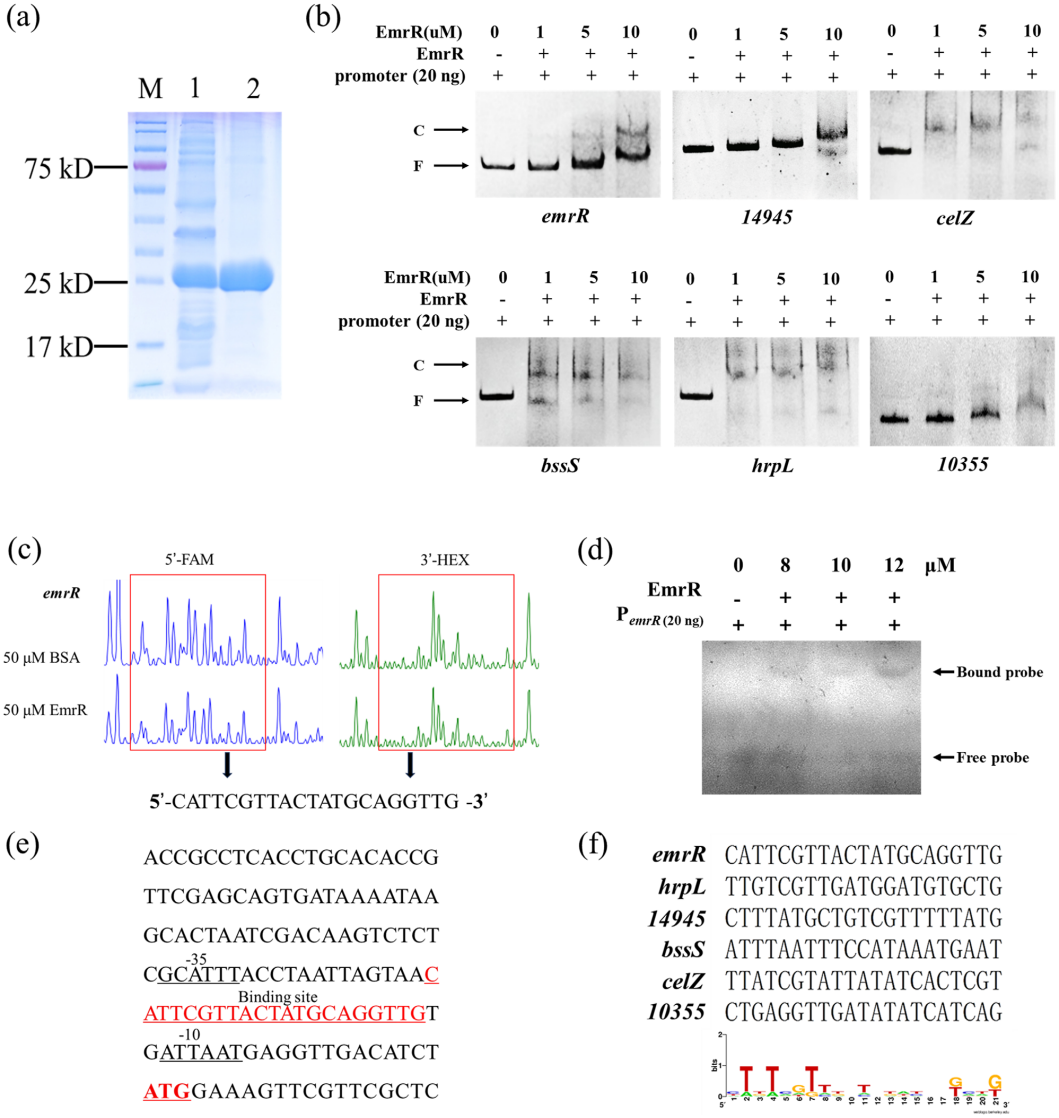
EmrR directly interacts with the promoters of *emrR*, *celZ*, *bssS*, *15945*, *hrpL* and *10355*. (a) Detection of purified EmrR protein. (b) Electrophoretic mobility shift assay was performed by the promoter DNA probes (20 ng) with 1, 5 or 10 μM EmrR. Free probe (F) and protein–DNA complexes (C) are indicated by arrows. The experiments were repeated at least three times. (c) DNase I footprinting was performed using EmrR and its promoter region of *emrR* labelled with 5′‐FAM and 3′‐HEX, respectively. (d) Electrophoretic mobility shift assay was performed to investigate the binding of EmrR to a 21‐bp DNA fragment, P_*emrR*_, which was identified through DNase I footprinting. Free probe and bound protein–DNA complexes are indicated by arrows. (e) Analysis of the upstream promoter region of *emrR*. (f) Identification of the conserved sites for EmrR binding by aligning the P*_emrR_* with the promoter sequences of P_*hrpL*_, P_*celZ*_, P_*bssS*_, P_*14945*_ and P_*10355*_. All experiments were repeated at least three times.

To investigate whether EmrR modulates transcriptional expression by interacting with the promoter regions of target genes, the EmrR protein was expressed using a prokaryotic expression system and subsequently purified (Figure 7a) for use in electrophoretic mobility shift assays (EMSA). The results demonstrated that EmrR at a final concentration of 1 μM, 5 μM or 10 μM induced a mobility shift in the promoter fragments of *emrR*, *14945*, *celZ*, *bssS*, *hrpL* and *10355* (Figure 7b), suggesting a direct interaction between EmrR and the corresponding promoters, P_*emrR*_, P_*14945*_, P_*celZ*_, P_*bssS*_, P_*hrpL*_ and P_*10355*_ to regulate the transcriptional expression of genes involved in c‐di‐GMP synthesis and degradation, cellulase production and the T3SS in *D. oryzae* .

To confirm the specific motif through which EmrR interacts with the promoter region, DNase I footprinting was conducted using the *emrR* promoter (P_*emrR*_) labelled with FAM (5′) and HEX (3′), following a previously described method (Liang et al. 2023). The results revealed a 21 bp motif (5′‐CATTCGTTACTATGCAGGTTG‐3′) within P_*emrR*_, designated as P_*emrR*(21)_, that was specifically bound by EmrR (Figure 7c). Subsequently, P_*emrR*(21)_ was validated using EMSA with purified EmrR protein and a synthesised P_*emrR*(21)_ probe. The EMSA results showed a distinct bound shift of the P_*emrR*(21)_ motif in the presence of EmrR (Figure 7d), confirming that this 21‐bp sequence serves as the specific binding motif for EmrR (Figure 7e).

Similarly, DNase I and binding site alignment analyses were conducted to identify the specific DNA binding motif of EmrR within the promoter regions of the genes *hrpL*, *celZ*, *bssS*, *14945* and *10355*. The results indicated that EmrR specifically binds to the following sequences: *hrpL* (5′‐TTGTCGTTGATGGATGTGCTG‐3′), *celZ* (5′‐TTATCGTATTATATCACTCGT‐3′), *bssS* (5′‐ATTTAATTTCCATAAATGAAT‐3′), *14945* (5′‐CTTTATGCTGTCGTTTTTATG‐3′) and *10355* (5′‐ACTGAGGTTGATATATCATC‐3′) (Figure 7f and Figure S8).

## 3 Discussion

MarR‐family transcriptional regulators play a central role in controlling stress responses, virulence and detoxification. Some MarR regulators act as both activators and repressors, depending on operator location relative to target promoters: binding upstream typically represses, whereas binding overlapping or downstream can activate genes (Deochand and Grove 2017). EmrR controls the multidrug‐resistance pump EmrAB in *E. coli* , *Chromobacterium violaceum* and *Agrobacterium tumefaciens* by binding to the *emrAB* promoter (Lomovskaya et al. 1995; Xiong et al. 2000; Barroso et al. 2018; Khemthong et al. 2019). However, the role of EmrR in *Dickeya* remains poorly characterised. In this study, we identified a conserved EmrR homologue in *D. oryzae* EC1 (Figures S1C and S2) and demonstrated that EmrR negatively regulates *emrRAB* (Figure 2) and critically modulates multiple virulence traits, including motility (Figure 1), cellulase production (Figure 3 and Figure S3), biofilm formation (Figure 4), T3SS gene expression and pathogenicity (Figure 6 and Figure S7). Collectively, these findings establish EmrR as a key regulator of virulence factor production, physiology and pathogenicity in *D. oryzae* EC1.

The *emrRAB* operon, encoding the multidrug‐efflux pump EmrAB, has been extensively studied in *E. coli* (Xiong et al. 2000) and is conserved in *Dickeya* (Figure S2). EmrAB consists of the major facilitator family transporter EmrB and the membrane fusion protein EmrA, which facilitates substrate transport across both membranes (Lomovskaya and Lewis 1992; Dinh et al. 1994). In *E. coli* , the repressor EmrR, encoded upstream of *emrAB*, negatively regulates the operon by binding an imperfect inverted repeat within the promoter (Lomovskaya et al. 1995). Similarly, in *Dickeya*, *emrR* is located directly upstream of *emrAB* (Figures S1 and S2), whereas in *Sinorhizobium meliloti* and *A. tumefaciens* it is transcribed divergently (Santos et al. 2014; Khemthong et al. 2019). The binding sites of EmrR vary among species: there is one site (5′‐CTGTCGTTACTATATCGGCTG‐3′) within the promoter in *E. coli* (Xiong et al. 2000), whereas two sites (one upstream of *emrR* and another within *emrA*) are present in *A. tumefaciens* (Khemthong et al. 2019). In this study, we identified the *emrRAB* operon in *D. oryzae* and demonstrated that EmrR directly binds to a specific site (5′‐CATTCGTTACTATGCAGGTTG‐3′) within its promoter region to repress transcription (Figures 2 and 7), as supported by β‐galactosidase activity assays, RT‐qPCR, EMSA and DNase I footprinting (Figures 2 and 7). These results indicate that EmrR is conserved in *Dickeya* species and employs a repressor mechanism similar to that in *E. coli* , despite species‐specific differences in binding sequences.

Biofilm formation and motility are crucial for bacterial colonisation and pathogenicity (Heindl et al. 2014; Nan and Zusman 2016; Hathroubi et al. 2018; Khan et al. 2020). We previously demonstrated that deleting all c‐di‐GMP phosphodiesterase (PDE) genes in *D. oryzae* EC1 abolishes motility, suppresses biofilm formation and blocks rice seed invasion (Chen et al. 2020). Here, ∆*emrR* significantly decreased swimming and swarming motility as well as biofilm formation (Figures 1 and 4). c‐di‐GMP globally regulates bacterial behaviours, particularly biofilm formation and motility, in many pathogens (Ryjenkov et al. 2006; Hengge 2009; Boehm et al. 2010; Chen et al. 2016). Its intracellular level is balanced by diguanylate cyclases (DGCs; containing GGDEF domains), which synthesise c‐di‐GMP from GTP and PDEs (with EAL or HD‐GYP domain), which degrade it (Boehm et al. 2010; He et al. 2007; Ryjenkov et al. 2006). In *Dickeya*, key c‐di‐GMP enzymes have been characterised: EcpB and EcpC (PDEs) in *D. dadantii* 3937 (Yi et al. 2010) and one DCG (*W909_14945*) and two PDEs (*W909_10355*, *W909_14950*) in *D. oryzae* EC1 (Chen et al. 2016, 2020). Recently, OhrR was shown to directly bind to the promoter region of these genes, upregulating *W909_14945* and downregulating *W909_10355*, thereby modulating c‐di‐GMP levels (Lv, Chen, et al. 2022; Lv, Ye, et al. 2022).

Riboswitches with high affinity for c‐di‐GMP enable reliable intracellular detection and regulate transcription and RNA splicing (Sudarsan et al. 2008). In this study, Δ*emrR* reduced c‐di‐GMP levels, as measured using a *vc2* (riboswitch)‐*lacZ* fusion (Figure 4d), correlating with downregulation of the DGC gene *W909_14945* and upregulation of the PDE genes *W909_10355* and *W909_14950* (Figure 4c). Because c‐di‐GMP generally promotes biofilm formation and inhibits motility, the observed reduction in motility in Δ*emrR* was unexpected, suggesting a c‐di‐GMP independent or integrated regulatory pathway. Notably, OhrR similarly modulates c‐di‐GMP in *D. oryzae* by repressing *W909_14945* and activating *W909_10355* and *W909_14950* (Lv, Chen, et al. 2022; Lv, Ye, et al. 2022), suggesting a conserved regulatory logic shared by EmrR.

Studies have shown that c‐di‐GMP suppresses T3SS gene expression in *D. dadantii* (Yi et al. 2010; Yuan et al. 2018), and low c‐di‐GMP levels correlate with increased virulence dependent on T3SS effectors in pathogens, such as *E. coli* and *Salmonella enterica* (Lamprokostopoulou et al. 2010; Moscoso et al. 2011; Zheng et al. 2013; Jiang et al. 2022). In the HrpX/Y‐HrpS‐HrpL pathway, HrpX/Y activates *hrpS*; HrpS then recruits RpoN‐RNA polymerase to transcribe *hrpL* (Chatterjee et al. 2002; Yap et al. 2005; Tang et al. 2006). In our study, deletion of the c‐di‐GMP synthase gene *W909_14945* lowered intracellular c‐di‐GMP and upregulated T3SS genes *hrpA* and *hrpN*, but not *hrpL*, as confirmed by β‐galactosidase activity assays (Figure 6a). Thus, reduced c‐di‐GMP enhance some T3SS genes without activating the master regulator *hrpL*. Similarly, ∆*emrR* showed stronger upregulation of T3SS genes compared to the wild‐type strain EC1 and the complemented strain ∆*emrR*(pLAFR‐*emrR*), as evidenced by RT‐qPCR and β‐galactosidase activity assays (Figure 6b,c). We further demonstrated that EmrR directly binds the *hrpL* promoter to repress its transcription (Figure 7). Additionally, swimming motility, biofilm formation, and potato slice maceration were significantly increased in ∆*hrpL*, ∆*hrpS* and ∆*hrpN* (Figure S7), confirming that EmrR represses the T3SS by downregulating *hrpL* transcription. The increased motility, enhanced biofilm formation, and attenuated virulence observed in ∆*emrR* likely result from synergistic repression of T3SS genes by EmrR and c‐diGMP, which outweighs the stimulatory effect of low c‐di‐GMP alone on motility. In contrast, previous research on *D. zeae* MS2 demonstrated that deleting T3SS (e.g., *hrpL* or *dsp*/*hrp*/*hrc* clusters) did not affect extracellular pectinase production but markedly reduced virulence (Hu et al. 2022). Such differences may reflect biological variation among *Dickeya* strains: *D. oryzae* EC1 encodes a complete T3SS and produces zeamines, whereas MS2 lacks zeamine synthesis.

In *Pseudomonas syringae* , the extracytoplasmic function sigma factor AlgU acts as a global virulence regulator, activating T3SS effector genes while suppressing flagella‐dependent motility (Markel et al. 2016; Bao et al. 2020; Huang et al. 2022). Similarly, in *Erwinia amylovora* , HrpL represses flagellar genes under *hrp*‐inducing conditions (Cesbron et al. 2006). In *D. oryzae* , the *emrR* deletion mutant exhibited increased T3SS expression and reduced motility. Although T3SS regulation is documented, the roles of individual T3SS genes in *D. oryzae* remain unclear. Here, we show that mutants of T3SS genes displayed enhanced swimming motility, biofilm formation and potato slice maceration, suggesting that the induced expression of T3SS can suppress other virulence traits, which is a strategy that may help minimise host immune activation during infection. These findings provide a foundation for dissecting T3SS functions in *D. oryzae* .

**FIGURE 8:**
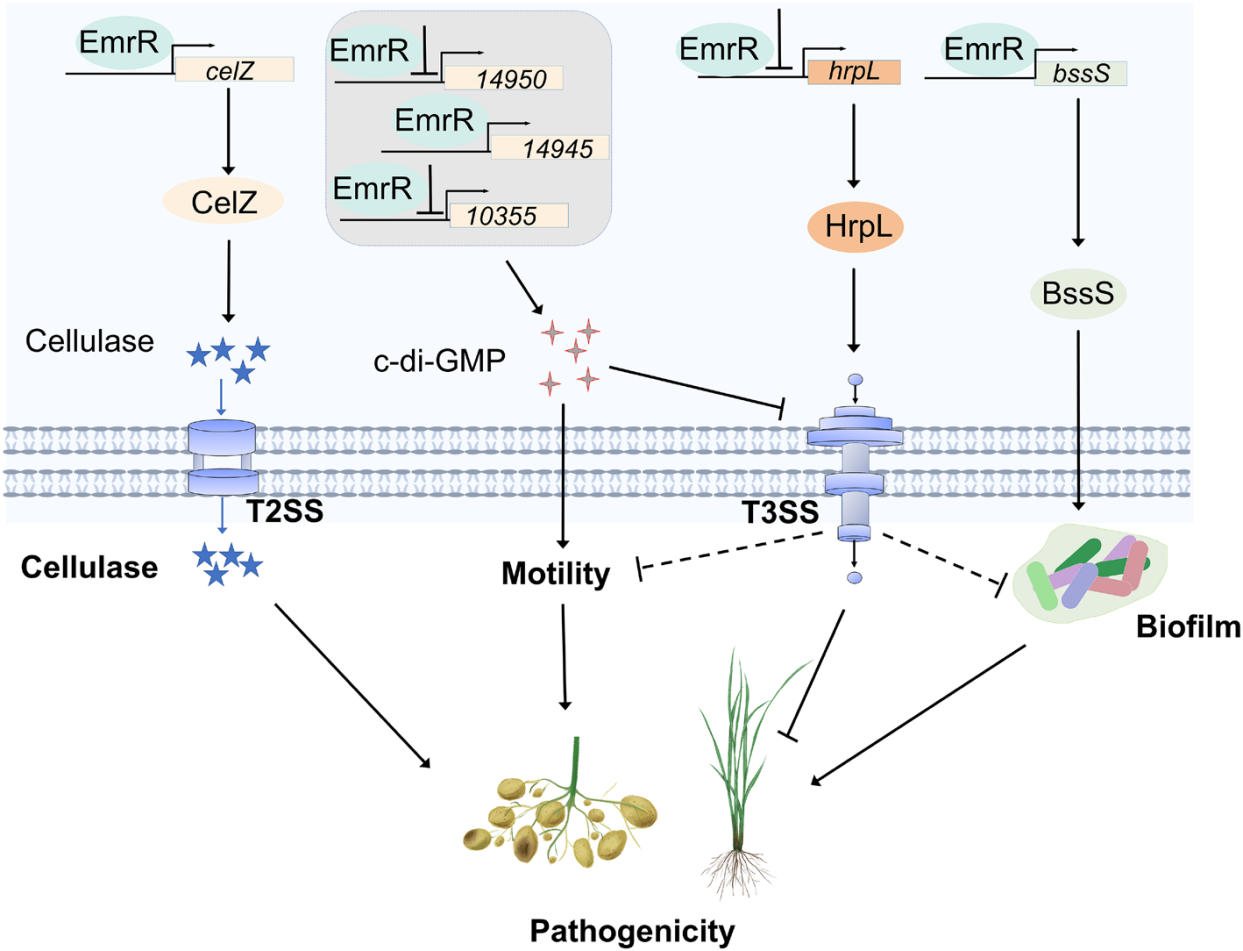
Schematic representation of the EmrR regulatory pathway in *Dickeya oryzae*. EmrR positively regulates the production of cellulase and biofilm formation by binding to the promoter region of *celZ* and *bssS*, respectively. Furthermore, EmrR negatively modulates the expression of type III secretion system (T3SS) genes through direct interaction with the promoter of *hrpL*, and mediates the levels of c‐di‐GMP. Specifically, EmrR enhances the expression of *W909_14945*, which encodes a c‐di‐GMP synthase, while simultaneously suppressing the expression of *W909_10355* and *W909_14950*, which encode c‐di‐GMP degradation enzymes.

In summary, this study demonstrates that the conserved transcriptional regulator EmrR in *Dickeya* is responsible for regulating the *emrRAB* operon, biofilm formation, cellulase production, motility and virulence. EmrR directly interacts with the promoter of T3SS regulator *hrpL* and regulates T3SS expression, additionally by modulating intracellular c‐di‐GMP levels (Figure 8). A conserved EmrR‐binding sequence was identified in the promoters of target genes. This study enhances the understanding of the MarR family regulation and provides potential avenues for the development of strategies to control *Dickeya* pathogenicity.

## 4 Experimental Procedures

### 4.1 Bacteria Strains and Growth Condition

The bacteria strains and plasmids used in this investigation are documented in Table 1. *D. oryzae* EC1 and its derivatives were cultivated at 28°C using Luria Bertani (LB) medium, minimal medium (MM) (Hussain et al. 2008), LS5 medium (Liao et al. 2014) or SOBGS medium (containing 20 g tryptone, 5 g yeast extract, 2.4 g MgSO_4_, 0.5 g NaCl, 0.186 g KCl and 10 g sucrose per litre) (Chen et al. 2020), as specified. *Escherichia coli* was grown at 37°C in LB medium. The medium was supplemented with the following final antibiotic concentrations, as required: streptomycin (Str), 50 μg/mL; polymyxin B sulphate (PB), 25 μg/mL; ampicillin (Amp), 100 μg/mL; kanamycin (Kan), 50 μg/mL and tetracycline (Tet), 15 μg/mL.

**TABLE 1:** Strains and plasmids used in this study.

Strains or plasmids	Relevant phenotypes and characteristics^a^	Source or reference
*Strains*		
EC1	Wild type of *Dickeya oryzae*, PB^r^	Hussain et al. (2008)
EC1_Δ*lacZ*_	*lacZ* in‐frame deletion mutant derived from EC1, PB^r^	This research
Δ*emrR*	*emrR* in‐frame deletion mutant derived from EC1, PB^r^	This research
Δ*emrR*_Δ*lacZ*_	*emrR* in‐frame deletion mutant derived from EC1_Δ*lacZ*_, PB^r^	This research
Δ*emrR*(pLAFR‐*emrR*)	Transformed the *emrR* mutant with plasmid pLAFR3 carrying the gene *emrR*, PB^r^, Tet^r^	This research
Δ*emrR*(pLAFR)	Transformed the *emrR* mutant with plasmid pLAFR3, PB^r^, Amp^r^	This research
EC1_Δ*lacZ*_ (pLME‐*emrR*)	Transformed the EC1_Δ*lacZ*_ with plasmid pLME‐*emrR*, PB^r^, Amp^r^	This research
Δ*emrR*_Δ*lacZ*_ (pLME‐*emrR*)	Transformed the Δ*emrR*_Δ*lacZ*_ with plasmid pLME‐*emrR*, PB^r^, Amp^r^	This research
EC1_Δ*lacZ*_ (pLME‐*emrAB*)	Transformed the EC1_Δ*lacZ*_ with plasmid pLME‐*emrAB*, PB^r^, Amp^r^	This research
Δ*emrR*_Δ*lacZ*_ (pLME‐*emrAB*)	Transformed the Δ*emrR*_Δ*lacZ*_ with plasmid pLME‐*emrAB*, PB^r^, Amp^r^	This research
Δ*hrpA*	*hrpA* in‐frame deletion mutant derived from EC1, PB^r^	This research
Δ*hrpN*	*hrpN* in‐frame deletion mutant derived from EC1, PB^r^	This research
Δ*hrpS*	*hrpS* in‐frame deletion mutant derived from EC1, PB^r^	This research
Δ*hrpL*	*hrpL* in‐frame deletion mutant derived from EC1, PB^r^	This research
EC1_Δ*lacZ*_ (pLME‐vc2)	Transformed the EC1_Δ*lacZ*_ with plasmid pLME‐vc2, PB^r^, Amp^r^	This research
Δ*14945*_Δ*lacZ*_	*lacZ* and 14945 in‐frame double deletion mutant derived from EC1, PB^r^	This research
Δ*14945*_Δ*lacZ*_ (pLME‐vc2)	Transformed the Δ*14945*_Δ*lacZ*_ with plasmid pLME‐vc2, PB^r^, Amp^r^	This research
EC1_Δ*lacZ*_ (pLME‐*hrpA*)	Transformed the EC1_Δ*lacZ*_ with plasmid pLME‐*hrpA*, PB^r^, Amp^r^	This research
EC1_Δ*lacZ*_ (pLME‐*hrpN*)	Transformed the EC1_Δ*lacZ*_ with plasmid pLME‐*hrpN*, PB^r^, Amp^r^	This research
EC1_Δ*lacZ*_ (pLME‐*hrpL*)	Transformed the EC1_Δ*lacZ*_ with plasmid pLME‐ *hrpL*, PB^r^, Amp^r^	This research
Δ*emrR*_Δ*lacZ*_ (pLME‐*hrpA*)	Transformed the Δ*emrR*_Δ*lacZ*_ with plasmid pLME‐*hrpA*, PB^r^, Amp^r^	This research
Δ*emrR*_Δ*lacZ*_ (pLME‐*hrpN*)	Transformed the Δ*emrR*_Δ*lacZ*_ with plasmid pLME‐*hrpN*, PB^r^, Amp^r^	This research
Δ*emrR*_Δ*lacZ*_ (pLME‐*hrpL*)	Transformed the Δ*emrR*_Δ*lacZ*_ with plasmid pLME‐*hrpL*, PB^r^, Amp^r^	This research
Δ*14945*_Δ*lacZ*_ (pLME‐*hrpA*)	Transformed the Δ*14945*_Δ*lacZ*_ with plasmid pLME‐*hrpA*, PB^r^, Amp^r^	This research
Δ*14945*_Δ*lacZ*_ (pLME‐*hrpN*)	Transformed the Δ*14945*_Δ*lacZ*_ with plasmid pLME‐*hrpN*, PB^r^, Amp^r^	This research
Δ*14945*_Δ*lacZ*_ (pLME‐*hrpL*)	Transformed the Δ*14945*_Δ*lacZ*_ with plasmid pLME‐*hrpL*, PB^r^, Amp^r^	This research
DH5α	*Escherichia coli* strain as host for plasmid constructs derived from pBBR1‐MCS4	Zhou et al. (2011)
HB101 (pRK2013)	*thr leu thi recA hsdR hsdM pro*, Kan^r^	Zhou et al. (2011)
CC118λ	*E. coli* strain as host for plasmid constructs derived from pKNG101	Zhou et al. (2011)
*Plasmids*		
pKNG101	Knockout vector, Str^r^	Zhou et al. (2011)
pLAFR3	Expression vector contains a *lacZ* promoter, Tet^r^	Song et al. (2020)
pLAFR‐*emrR*	pLAFR3 carries the coding region of *emrR* downstream of *lacZ* promoter, Tet^r^	This research
pLME	A vector containing with the non‐promoter was modified based on the pBBR1‐MCS4 backbone, Amp^r^	This research
pLME‐*emrR*	pLME of non‐promoter containing *lacZ* carries the promoter region of *emrR*, Amp^r^	This research
pLME‐*emrAB*	pLME of non‐promoter containing *lacZ* carries the promoter region of *emrAB* operon, Amp^r^	This research
pLME‐*hrpA*	pLME of non‐promoter containing *lacZ* carries the promoter region of *hrpA*, Amp^r^	This research
pLME‐*hrpN*	pLME of non‐promoter containing *lacZ* carries the promoter region of *hrpN*, Amp^r^	This research
pLME‐*hrpL*	pLME of non‐promoter containing *lacZ* carries the promoter region of *hrpL*, Amp^r^	This research
pKNG101‐*emrR*	pKNG101 carries the in‐frame deleted fragment of *emrR*, Str^r^	This research
pET30a‐*emrR*	pET30a carries the *emrR* coding region, Kan^r^	This research

^a^ PB^r^, Amp^r^, Kan^r^, Str^r^, Tet^r^ = resistance to polymyxin B sulfate, ampicillin, kanamycin, streptomycin or tetracycline, respectively.

### 4.2 Mutant Construction and Complementation

To generate deletion mutants, the flanking sequences of target genes were amplified by PCR using primers 1 and 2, 3 and 4(Table S1). The amplified DNA fragments were then purified using the Universal DNA purification Kit (Tiangen Biotech Co.). Subsequently, the purified DNA fragments were fused to the suicide vector pKNG101 that had been digested with BamHI and SpeI, using the ClonExpress MultiS kit (Vazyme Biotech Co.). The resulting recombinant vectors were transformed into competent cells of *E. coli* CC118λ and introduced into the wild‐type strain EC1 through triparental conjugation following a previously described method (Lv, Chen, et al. 2022; Lv, Ye, et al. 2022). Finally, confirmation of the deletion mutant was performed via PCR analysis employing primers F and R.

The coding sequence of the target gene was amplified by PCR and purified using the Universal DNA purification Kit (Tiangen Biotech Co.) for construction of vectors to complement deletion mutation strains. The purified DNA fragments were then ligated into pLAFR3, which had been digested with BamHI and HindIII, using the ClonExpress MultiS kit. Subsequently, the constructed vector was transformed into *E. coli* DH5α and introduced into corresponding mutants through triparental conjugation following a previously described method (Lv, Chen, et al. 2022; Lv, Ye, et al. 2022). Finally, confirmation of complemented strains was confirmed by PCR using primers pLAFR‐F and pLAFR‐R.

### 4.3 Construction of c‐di‐GMP Reporter Plasmid

The c‐di‐GMP levels in wild‐type strain and derivative strains from *D. oryzae* were assessed. A modified version plasmid pBBRI‐MCS4, named pLME, was used as a c‐di‐GMP reporter plasmid by incorporating a promoterless *lacZ* gene. To construct this reporter plasmid, pLME was linearised with BamHI, and a synthesised sequence of vc2 (Sudarsan et al. 2008) containing the homologous region flanked by BamHI sites on both ends was inserted into pLME using the ClonExpress MultiS kit. The resulting plasmid pLME‐*vc2* was then transformed into wild‐type strain EC1 and its derivatives through triparental conjugation. Finally, the β‐galactosidase activity was determined using the Miller method.

### 4.4 Quantitative β‐Galactosidase Activity Assay

The β‐galactosidase activity was determined following a previously described method (Sudarsan et al. 2008). Briefly, the pellet of bacterial cultures (1 mL) was resuspended in an equal volume of prechilled Z buffer (Na_2_HPO_4_ 0.06 M, NaH_2_PO_4_ 0.04 M, KCl 0.01 M, MgSO_4_ 1 mM, β‐mercaptoethanol 0.05 M). Subsequently, a resuspension of cells (100 μL) was mixed with Z buffer (900 μL) for determining the optical density at 600 nm (OD_600_). To permeabilise another set of cells (100 μL), SDS solution (0.05%, 20 μL), chloroform (20 μL), and additional Z buffer were added followed by vigorous vortexing for 20 s and incubation for 20 min at 30°C. The reaction was initiated by adding ONPG solution (*o*‐nitrophenyl‐β‐D‐galactopyranoside; final concentration: 0.4%) to the mixture and allowed to proceed without specifying the duration time (*t*). Once sufficient yellow colour developed, the reaction was stopped by adding Na_2_CO_3_ solution (1 M; 250 μL). Finally, each sample was centrifuged for 10 min to remove debris and chloroform before measuring the optical density of the supernatant at 420 nm. The Miller Units were calculated as follows: Miller Units = (OD_420_ × 1000)/(OD_600_ × *t*), where *t* is the time in minutes of the ONPG reaction.

### 4.5 Bacterial Growth Kinetics Assay

The bacterial cultures were grown overnight in LB medium, and then adjusted to the same cell density by being diluted in the LB and LS5 medium. Subsequently, 800 μL of each diluted culture were added to the 2 mL centrifuge tubes and incubated at 28°C under identical conditions. The density of bacterial cells was measured every 3 h, with each strain repeated three times at each time point in triplicate.

### 4.6 Zeamine Production Bioassay

The bioassay for zeamine production was conducted on LS5 medium following the previously described protocol (Lv, Chen, et al. 2022; Lv, Ye, et al. 2022). Zeamine concentrations were determined using the formula: Zeamine (unit) = 0.5484e^0.886*x*^, where *R* ^2^ (correlation coefficient) is 0.9957 and *x* represents the radius in millimetres of the inhibition zone surrounding the well (Zhou et al. 2011; Cheng et al. 2013; Liao et al. 2014; Chen et al. 2016; Lv et al. 2018; Lv, Chen, et al. 2022; Lv, Ye, et al. 2022).

### 4.7 Extracellular Enzyme Activity Assay

Carboxymethyl cellulose sodium, polygalacturonic acid, and skimmed milk were used as the substrates for the bioassay plates. The activities of cellulase, pectate lyase and protease were measured separately using these substrates (Chatterjee et al. 1995; Caldas et al. 2002; Lv et al. 2018). To prepare the extracellular enzymes bioassay plates, a volume of 35 mL medium was poured into 120 × 120 mm plates. After solidification, wells with a diameter of 5 mm were punched. These wells were then filled with supernatants obtained from bacterial cultures that had reached an optical density at OD_600_ = 1.3 and subsequently centrifuged at 13,000 *g* for 5 min. The bioassay plates were incubated at 28°C for a duration of 14–20 h, following this incubation period, the cellulase plates were stained using a solution containing Congo Red at a concentration of 0.1% (wt/vol) and decolourised using 1 M NaCl. The pectate lyase plates were treated with 1 M HCl while the transparent zones on the protease assay plates were observed.

### 4.8 Biofilm Formation Assay

The biofilm formation assay was conducted according to the previously described protocol (Chen et al. 2020). An overnight bacterial culture was diluted 1:1000 in SOBG medium and then transferred to 10‐mL glass tubes at a volume of 2 mL. The tubes were incubated at 28°C for 24 h without agitation. Subsequently, the cultures were gently poured off and washed with water at least three times. The bacterial biofilm mass was stained with 2 mL 0.1% (wt/vol) crystal violet solution for 15 min. To eliminate any unbound dye, the tubes were rinsed three times with water. For quantification of the biofilm mass, the stained cells were decoloured using 70% ethanol after drying, and their absorbance at a wavelength of 570 nm was measured.

### 4.9 Flagellum‐Mediated Motility Assay

The swimming motility was evaluated using a semisolid medium plate (10 g Bacto tryptone, 5 g NaCl and 2 g Bacto agar per litre). A 1 μL aliquot of the overnight bacterial culture was inoculated at the centre of the assay plate and incubated at 28°C for 14–24 h. Subsequently, the diameter of bacterial spread was measured. The collective swarming motility of bacteria was assessed on semisolid medium plates (10 g tryptone, 5 g NaCl and 4 g agarose per litre).

### 4.10 Pathogenicity Assay for Rice Seeds Germination

The rice seed germination assay was conducted according to a previously described method (Chen et al. 2020). Briefly, overnight bacterial cultures were diluted in LB medium and incubated at 28°C with shaking at 200 rpm. The bacterial cultures were then centrifuged at 4°C with 5000 *g*, and the supernatant was removed, and the cells were resuspended with PBS (pH 7.0). The resuspended bacterial cultures were adjusted to an OD_600_ of 0.6 and further diluted in a series of eight‐fold dilutions using PBS. The bacterial colony‐forming units (cfu) were determined by performing a heterotrophic plate counting assay. Twenty rice seeds (Te Xianzhan, from the Rice Research Institute, Guangdong Academy of Agricultural Sciences, Guangzhou, China) were immersed into a volume of 10 mL of the diluted bacterial cultures for a duration of 6 h at room temperature. Subsequently, the rice seeds were transferred onto the plates with two moistened filter papers after being rinsed three times with sterile water. The plates were then incubated at 28°C under an alternating cycle of 8 h dark and 16 h light conditions with periodic supplementation of sterile water if necessary. As a control group, some rice seeds were treated with only PBS solution without any bacteria added. After a 7‐day incubation period, measurements including germination inhibition rate as well as root and shoot length of rice plantlets were recorded.

### 4.11 Pathogenicity Assay for Potato Slice Maceration

Potato tubers ( *Solanum tuberosum* ‘Bintje’) were air‐dried on a paper towel after being washed with tap water and sliced evenly to a thickness of approximately 5 mm. Subsequently, the potato slices were rinsed three times with sterile water and transferred onto two moistened filter papers placed on sterilised dishes, following a 30 min drying period on sterilised filter paper. Bacterial cells (1 μL) of OD_600_ = 1.0 in LB medium were inoculated at the centre of each potato slice, which was then incubated at 27°C for 24 and 48 h. Symptom development and measurements of the potato tubers were observed throughout the experiment, which was repeated at least three times with triplicates.

### 4.12 RNA Extraction and RT‐qPCR Analysis

For total RNA isolation, *D. oryzae* EC1 and the *emrR* mutant MM cultures were centrifuged, and the total RNA was extracted using the SV total RNA isolation system kit (Promega), following the manufacturer's instructions. To eliminate genomic DNA contamination, the RNA samples were treated with DNase I (Takara) at 37°C for 1 h, subsequently purified using an RNA Clean Kit, and verified via PCR amplification with the 16S rRNA primer pair. RNA integrity was assessed by agarose gel electrophoresis, and its concentration was quantified using a UV5 Nano spectrometer (Mettler Toledo).

For RT‐qPCR analysis, equal quantities of RNA from EC1 and *emrR* mutant were reverse transcribed into cDNA using the StarScript pro All‐in‐one RT Mix (GenStar). Subsequently, qPCR was performed using SuperReal PreMix Plux (SYBR Green, 2×) (Tiangen Biotech Co. Ltd) on a QuantStudio 6 Flex Real‐Time PCR system (Applied Biosystems), following the manufacturer's instructions. Primer specificity and efficiency were evaluated via melting curve analysis. Gene expression levels were calculated using the 2^−∆∆*C*t^ method, where ∆∆*C*_t_ was determined as Δ*C*_t_1 − Δ*C*_t_2, as described by Livak and Schmittgen (2001). 16S rRNA was used as the internal reference gene. The RT‐qPCR experiments were performed at least in duplicate, using RNA samples isolated from triplicate cultures in each run.

### 4.13 Electrophoretic Motility Shift Assay

The binding of EmrR to the DNA promoter region was evaluated using an electrophoretic mobility shift assay (EMSA), following previously established protocols (Lv et al. 2018, 2019). EmrR protein was purified from the pET30a expression system expressed in *E. coli* BL21 (DE). Binding reactions were conducted between EmrR (at final concentrations of 0, 1, 5 and 10 nM) and 20 ng of DNA promoter fragments. The coding region of *emrR* and DNA sequences of the target promoter regions were amplified by PCR using primers listed in Table S1.

### 4.14 DNase I Footprinting

The DNase I footprinting was conducted according to a previously described method with minor modifications (Cha et al. 2019). The promoter region of *emrR* operon was amplified using primer pairs P*emrR*‐F/P*emrR*‐R labelled with FAM and HEX, respectively. The probes of FAM/HEX‐labelled were purified and quantified using a UV5 Nano spectrometer (Mettler Toledo). For the DNase assay, 400 ng DNA probes were incubated with either 50 μM EmrR or 50 μM bovine serum albumen (BSA) for 30 min at 25°C in a 40 μL reaction system. Subsequently, 10 μL of a solution containing 0.03 units of DNase I (New England Biolabs) was added to each reaction mixture. The reaction was terminated by addition of 200 mM EDTA. Following termination, DNA was purified through phenol/chloroform extraction and resuspended in water to obtain a final volume of 25 μL. Finally, the DNA samples were analysed on 3730xl system and the data were processed using Peak Scanner software version 2 (Applied Biosystems).

### 4.15 Statistical Analysis

All experiment was performed in triplicates and independently repeated at least three times. In certain cases, mutant data were normalised to the wild‐type EC1, which was arbitrarily defined as 100%, to facilitate comparative analysis. Statistical analysis was conducted using Prism 8.0 software (GraphPad) by applying a paired two‐tailed Student's *t*‐test (ns, not significant *p* > 0.1, *p < 0.1, **p < 0.01, ***p < 0.001, ****p < 0.0001) or one‐way ANOVA (*p* < 0.05) to compare the wild‐type EC1 with its derivatives.

## Author Contributions

**Ziwei Meng:** investigation. **Wenqi Huang:** investigation. **Yingying Cheng:** investigation, writing – review and editing. **Xiaojing Fan:** methodology, software. **Xiaoyuan Chen:** resources, methodology. **Mingfa Lv:** writing – original draft, supervision. **Lianhui Zhang:** resources, methodology. **Peng Li:** methodology, resources. **Tao Zhuo:** methodology, investigation, software. **Zeling Xu:** resources, methodology. **Jianuan Zhou:** methodology, formal analysis. **Shaohua Chen:** software, methodology. **Ming Hu:** writing – review and editing, methodology.

## Funding

This work was supported by the grants from the National Natural Science Foundation of China (32570212, 32400145, U22A20480), the Science Foundation of Fujian Provincial Science & Technology Department (2023J01440, 2022J01128), Fujian Agriculture and Forestry University Science and Technology Innovation Special Foundation (KFb22015XA) and the Open fund of the Guangdong Provincial Key Laboratory of Utilization and Conservation of Food and Medicinal Resources in Northern Region (FMR2025004M).

## Conflicts of Interest

The authors declare no conflicts of interest.

## Supporting information


**Figure S1:** Gene arrangement of the *emrR*‐*emrAB* operon in *Dickeya*.

**Figure S2:** The evolutionary relationships of EmrR within the *Dickeya* genus.

**Figure S3:** Detection of pectinase and protease activity.

**Figure S4:** Determination of zeamine production in the wild‐type strain EC1 and its derivative strains.

**Figure S5:** Expression analysis of *hrpN*, *hrpA* and *hrpL*.

**Figure S6:** The swimming motility and maceration abilities of mutants ∆*hrpL*, ∆*hrpS*, ∆*hrpA* and ∆*hrpN* were evaluated using corresponding assays.

**Figure S7:** The maceration abilities assay on the potato slices.

**Figure S8:** DNase I footprinting analysis.

**Table S1:** Primers used in this study.

## Data Availability

The data that supports the findings of this study are available in the Supporting Information of this article.
