# Supplementary figures and images for: The MarR Family Transcriptional Regulator EmrR Negatively Regulates the Type III Secretion System (T3SS) and Positively Modulates Pathogenicity in Dickeya oryzae
Source: Mol Plant Pathol. 2026 Apr 6;27(4):e70255. doi: 10.1111/mpp.70255 (PMC13053672; doi:10.1111/mpp.70255)

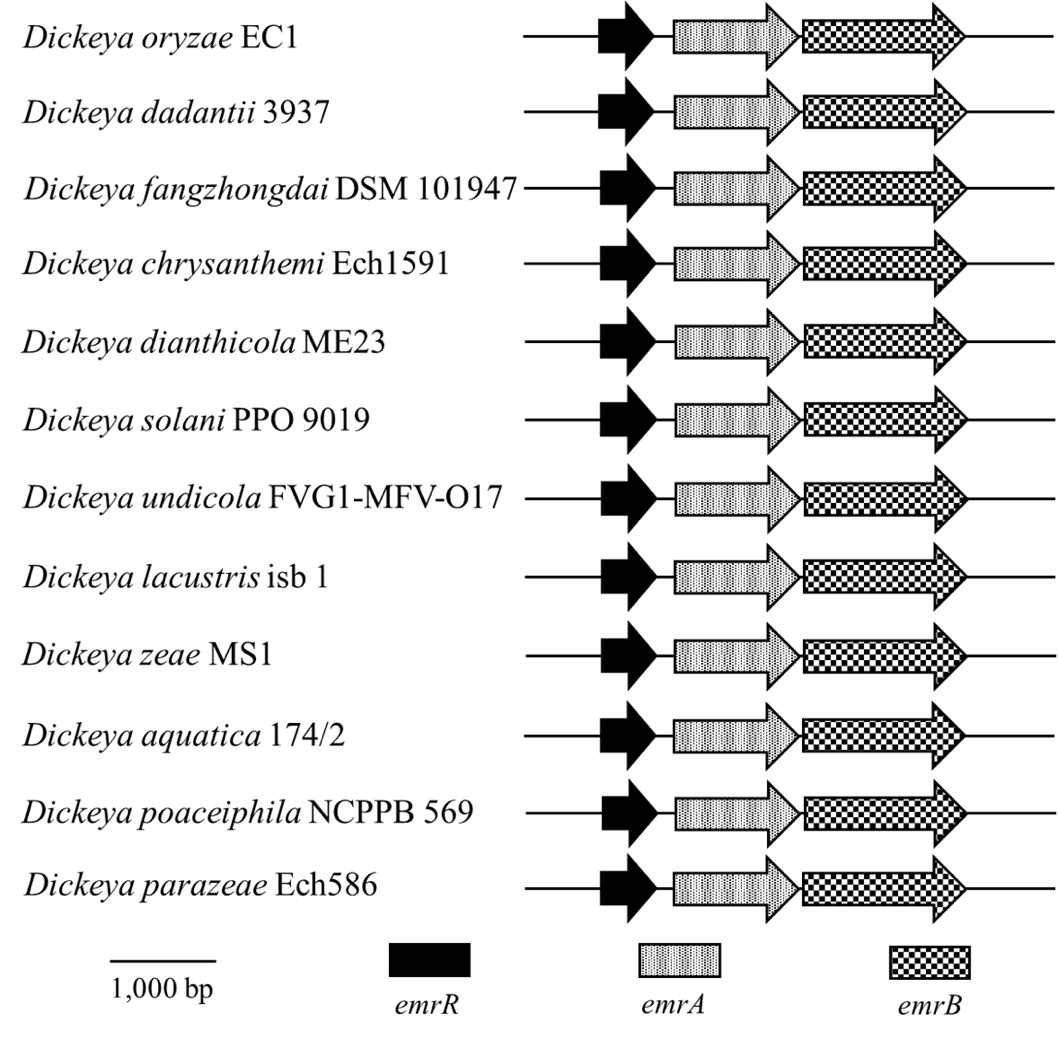


Figure S1. Gene arrangement of the *emrR*-*emrAB* operon in *Dickeya*.

Supplement: Supplementary file 1 — Figure S1: Gene arrangement of the emrR‐emrAB operon in Dickeya. [file MPP-27-e70255-s002.docx]
