# Supplementary material for: The MarR Family Transcriptional Regulator EmrR Negatively Regulates the Type III Secretion System (T3SS) and Positively Modulates Pathogenicity in Dickeya oryzae
Source: Mol Plant Pathol. 2026 Apr 6;27(4):e70255. doi: 10.1111/mpp.70255 (PMC13053672; doi:10.1111/mpp.70255)
Supplement: Supplementary file 2 — Figure S2: The evolutionary relationships of EmrR within the Dickeya genus. [file MPP-27-e70255-s005.docx]

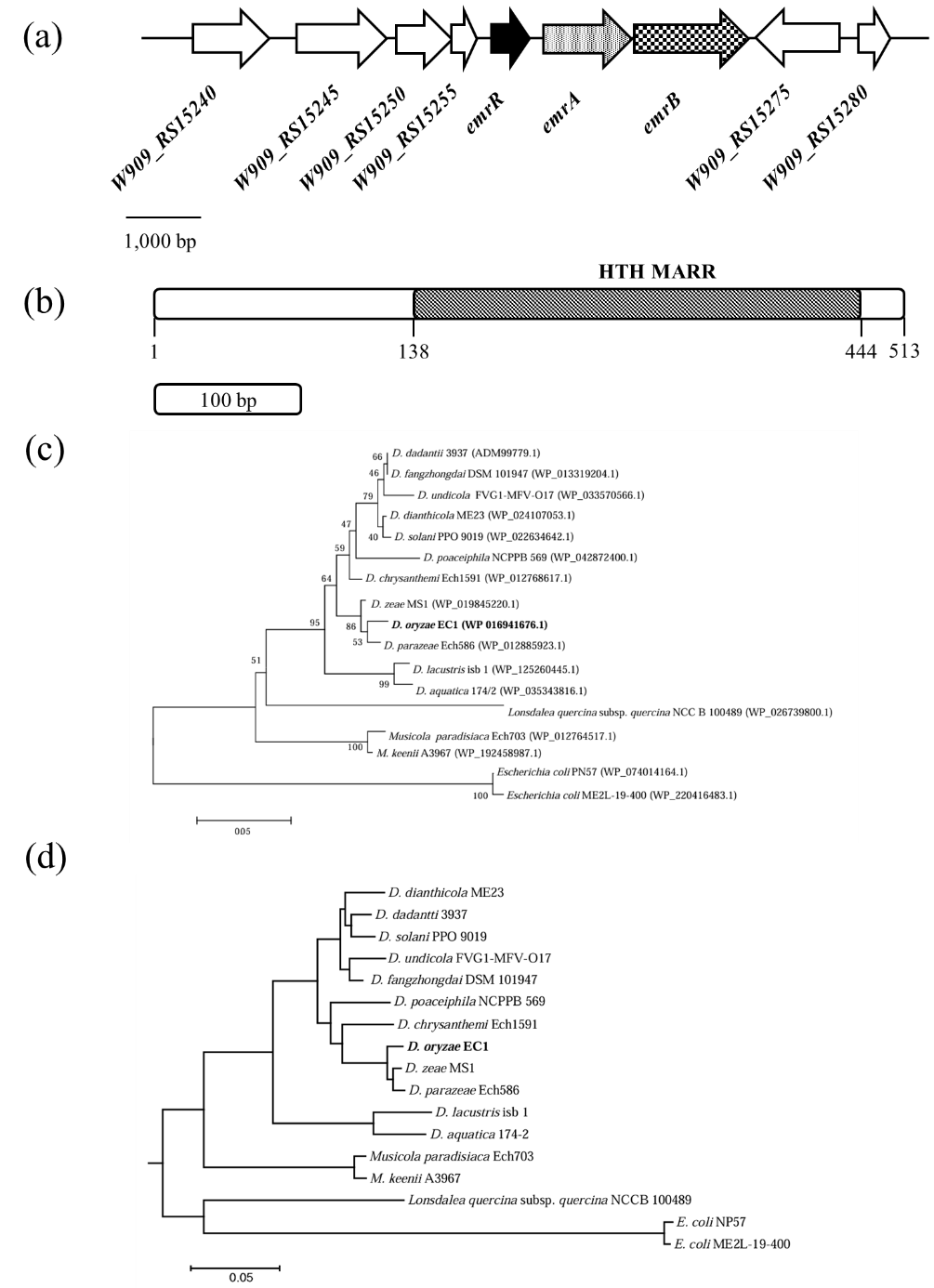


Figure S2. The evolutionary relationships of EmrR within the *Dickeya* genus. (a) The arrangement of *emrR*-*emrAB* in the *Dickeya oryzae* genome. (b) The domain of EmrR. (c) The phylogeny analysis of *emrR* in the *Dickeya*. (d) The phylogeny analysis of *Dickeya* genus. Using OrthoFinder v2.3.2 (Emms and Kelly, 2019), a total of 1,384 single-copy orthologous genes were identified across the genomes of the 17 strains, and these genes were used to construct a phylogenetic tree.

**Reference**

Emms DM, Kelly S. 2019. OrthoFinder: phylogenetic orthology inference for comparative genomics. *Genome Biology*. 1:238.
