# Supplementary material for: The MarR Family Transcriptional Regulator EmrR Negatively Regulates the Type III Secretion System (T3SS) and Positively Modulates Pathogenicity in Dickeya oryzae
Source: Mol Plant Pathol. 2026 Apr 6;27(4):e70255. doi: 10.1111/mpp.70255 (PMC13053672; doi:10.1111/mpp.70255)
Supplement: Supplementary file 3 — Figure S3: Detection of pectinase and protease activity. [file MPP-27-e70255-s001.docx]

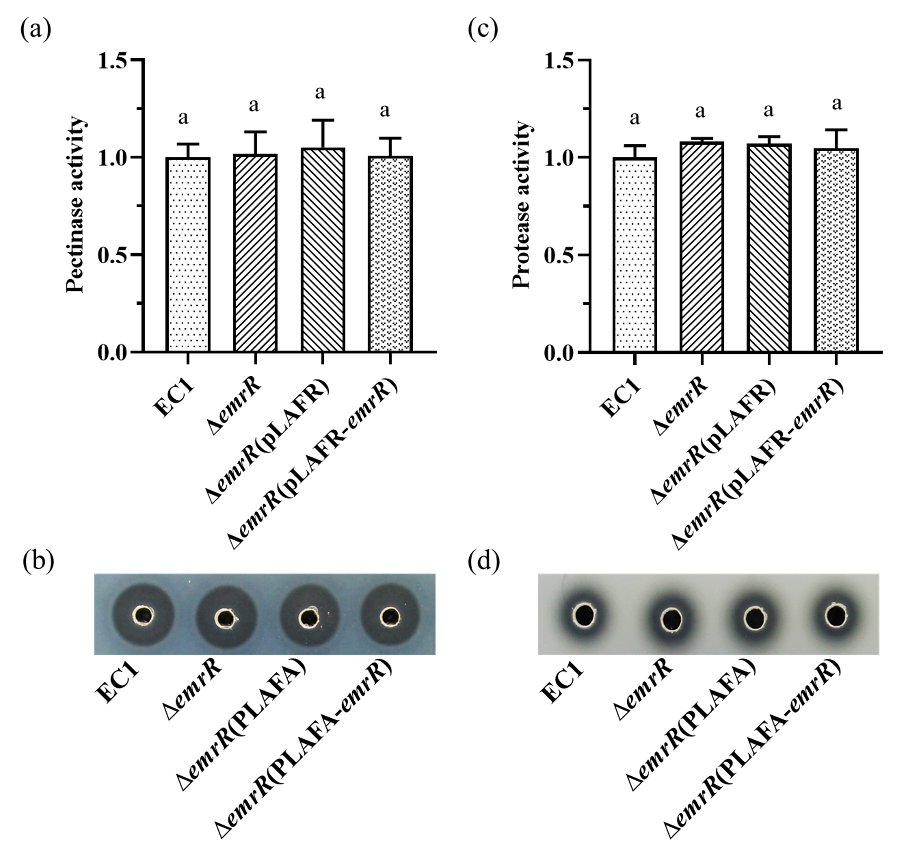


Figure S3. Detection of pectinase and protease activity. (a) Quantitative detection of extracellular pectinase activity in wild-type strain EC1 and its derivative strains. (b) Qualitatively detection the activity of pectinase on the enzyme activity assay plate. (c) Quantitative detection of extracellular protease activity in wild-type strain EC1 and its derivative strains. (d) Qualitatively detection the activity of protease on the enzyme activity assay plate. The final results of the mutant ∆*emrR* and its complemented strain ∆*emrR*(pLAFR-*emrR*) were normalized relative to those of the wild-type EC1, which was designated as one for comparative purpose (a and c). Experiments were repeated at least three times in triplicate and errors indicate stand deviation. Statistical analysis was carried out for each data group, and values that were significantly different (ANOVA, *p* < 0.05) are denoted by distinct letters.
