# Supplementary material for: The MarR Family Transcriptional Regulator EmrR Negatively Regulates the Type III Secretion System (T3SS) and Positively Modulates Pathogenicity in Dickeya oryzae
Source: Mol Plant Pathol. 2026 Apr 6;27(4):e70255. doi: 10.1111/mpp.70255 (PMC13053672; doi:10.1111/mpp.70255)
Supplement: Supplementary file 4 — Figure S4: Determination of zeamine production in the wild‐type strain EC1 and its derivative strains. [file MPP-27-e70255-s009.docx]

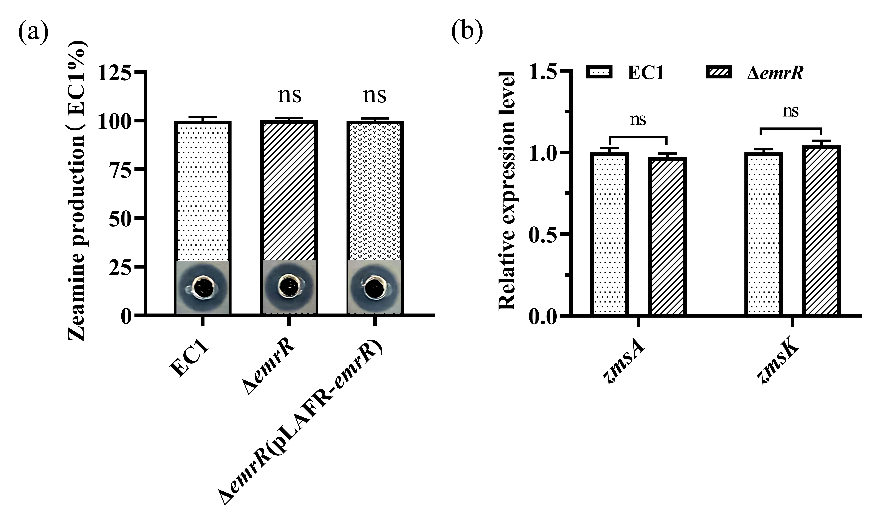


Figure S4. Determination of zeamine production in the wild-type strain EC1 and its derivative strains. (a) quantitative determination of zeamine production in strain EC1 and its derivatives. The concentration of zeamine was calculated using the following formular: zeamine (unit) = 0.5484e^0.886x^ (R^2^=0.9957), where x represents the radium in millimeters of the inhibition zone surrounding the well. For comparison purposes, the data for the mutant ∆*emrR* and its complemented strain ∆*emrR*(pLAFR-*emrR*) were normalized relative to that of the wild-type EC1, which was assigned a value of 100%. (b) Qualitative detection of zeamine production by EC1 and its derivatives using a bioassay plate. The antimicrobial activity bioassay plates were prepared as follows: 20 ml of 1% agarose containing approximately 10^8^ cells of *Escherichia coli* DH5α was overlaid onto 120 × 120 mm plates containing 15 ml LB agar. All experiments were conducted in triplicate and repeated three times independently. (c) Expression analysis of *zmsA* and *zmsK*, key genes involved in zeamine biosynthesis, was conducted using RT-qPCR. Statistical analysis was performed for each data group, and significant differences (ANOVA, *p* < 0.05) are indicated by different letters.
