# Supplementary material for: The MarR Family Transcriptional Regulator EmrR Negatively Regulates the Type III Secretion System (T3SS) and Positively Modulates Pathogenicity in Dickeya oryzae
Source: Mol Plant Pathol. 2026 Apr 6;27(4):e70255. doi: 10.1111/mpp.70255 (PMC13053672; doi:10.1111/mpp.70255)
Supplement: Supplementary file 5 — Figure S5: Expression analysis of hrpN, hrpA and hrpL. [file MPP-27-e70255-s008.docx]

Figure S5. Expression analysis of *hrpN*, *hrpA*, and *hrpL*. The expression levels of *hrpN*, *hrpA*, and *hrpL* in the mutant ∆*14945*, a strain deficient in a key gene involved in c-di-GMP biosynthesis, were evaluated using RT-qPCR. The experiment was performed in triplicate and i three times. Statistical significance was determined using Student’s *t*-test: ** *p* < 0.01, *** *p* < 0.0001.
