# Supplementary material for: The MarR Family Transcriptional Regulator EmrR Negatively Regulates the Type III Secretion System (T3SS) and Positively Modulates Pathogenicity in Dickeya oryzae
Source: Mol Plant Pathol. 2026 Apr 6;27(4):e70255. doi: 10.1111/mpp.70255 (PMC13053672; doi:10.1111/mpp.70255)
Supplement: Supplementary file 6 — Figure S6: The swimming motility and maceration abilities of mutants ∆hrpL, ∆hrpS, ∆hrpA and ∆hrpN were evaluated using corresponding assays. [file MPP-27-e70255-s006.docx]

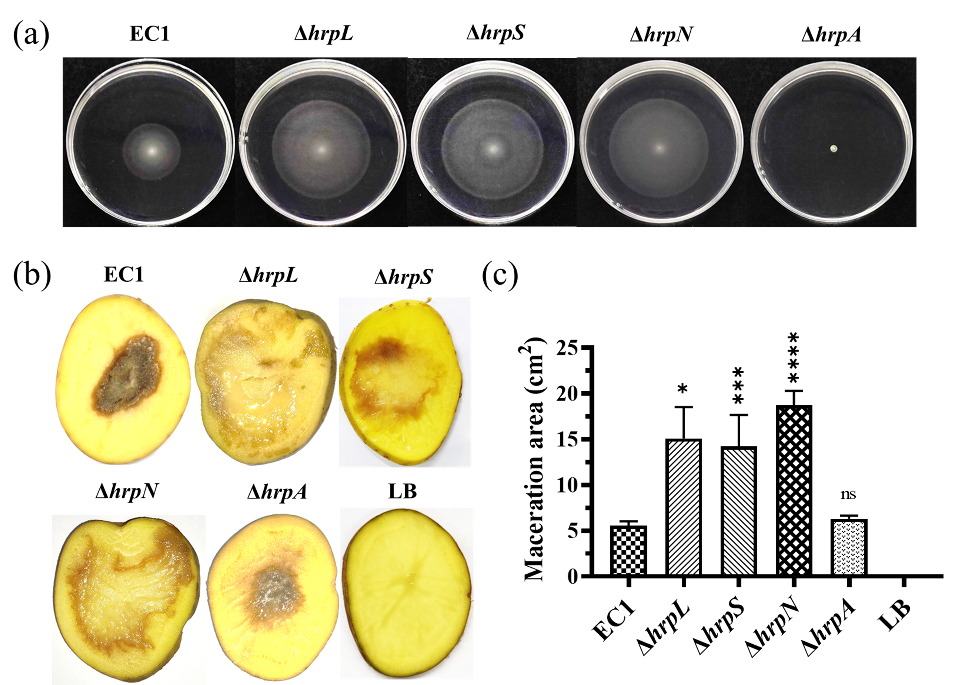


Figure S6. The swimming motility and maceration abilities of mutants ∆*hrpL*, ∆*hrpS*, ∆*hrpA*, and ∆*hrpN* were evaluated using corresponding assays. (a) The swimming motility assay for mutants ∆*hrpL*, ∆*hrpS*, ∆*hrpA*, ∆*hrpN*. (b) Maceration capacity of mutants ∆*hrpL*, ∆*hrpS*, ∆*hrpA*, and ∆*hrpN* on potato slices. (c) Evaluation of the maceration area by mutants ∆*hrpL*, ∆*hrpS*, ∆*hrpN*, and ∆*hrpA*. All experiments were conducted in triplicate and independently repeated at least three times, with the reported errors representing the standard deviation. * *p* < 0.1, *** *p* < 0.0001, **** *p* < 0.0001, Student's *t* test.
