# Supplementary material for: The MarR Family Transcriptional Regulator EmrR Negatively Regulates the Type III Secretion System (T3SS) and Positively Modulates Pathogenicity in Dickeya oryzae
Source: Mol Plant Pathol. 2026 Apr 6;27(4):e70255. doi: 10.1111/mpp.70255 (PMC13053672; doi:10.1111/mpp.70255)
Supplement: Supplementary file 7 — Figure S7: The maceration abilities assay on the potato slices. [file MPP-27-e70255-s007.docx]

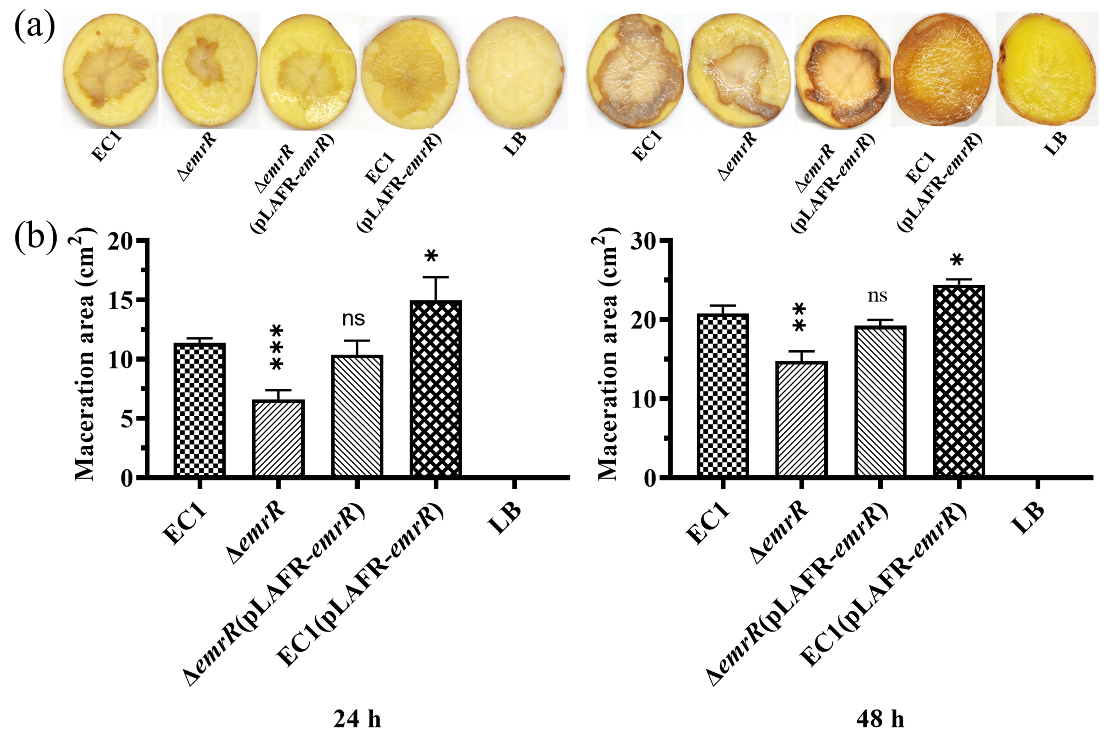


Figure S7. The maceration abilities assay on the potato slices. The maceration ability was assayed by measuring the maceration area on potato slices after 24 hours and 48 hours of incubation. (a) The maceration of visualizing on potato slices. (b) Statistics of maceration area on potato slices. The potato slices experiments were repeated at least three times in five and errors indicate stand deviation. * *p* < 0.1, *** *p* < 0.0001, Student's *t* test.
