# Supplementary material for: The MarR Family Transcriptional Regulator EmrR Negatively Regulates the Type III Secretion System (T3SS) and Positively Modulates Pathogenicity in Dickeya oryzae
Source: Mol Plant Pathol. 2026 Apr 6;27(4):e70255. doi: 10.1111/mpp.70255 (PMC13053672; doi:10.1111/mpp.70255)
Supplement: Supplementary file 8 — Figure S8: DNase I footprinting analysis. [file MPP-27-e70255-s003.docx]

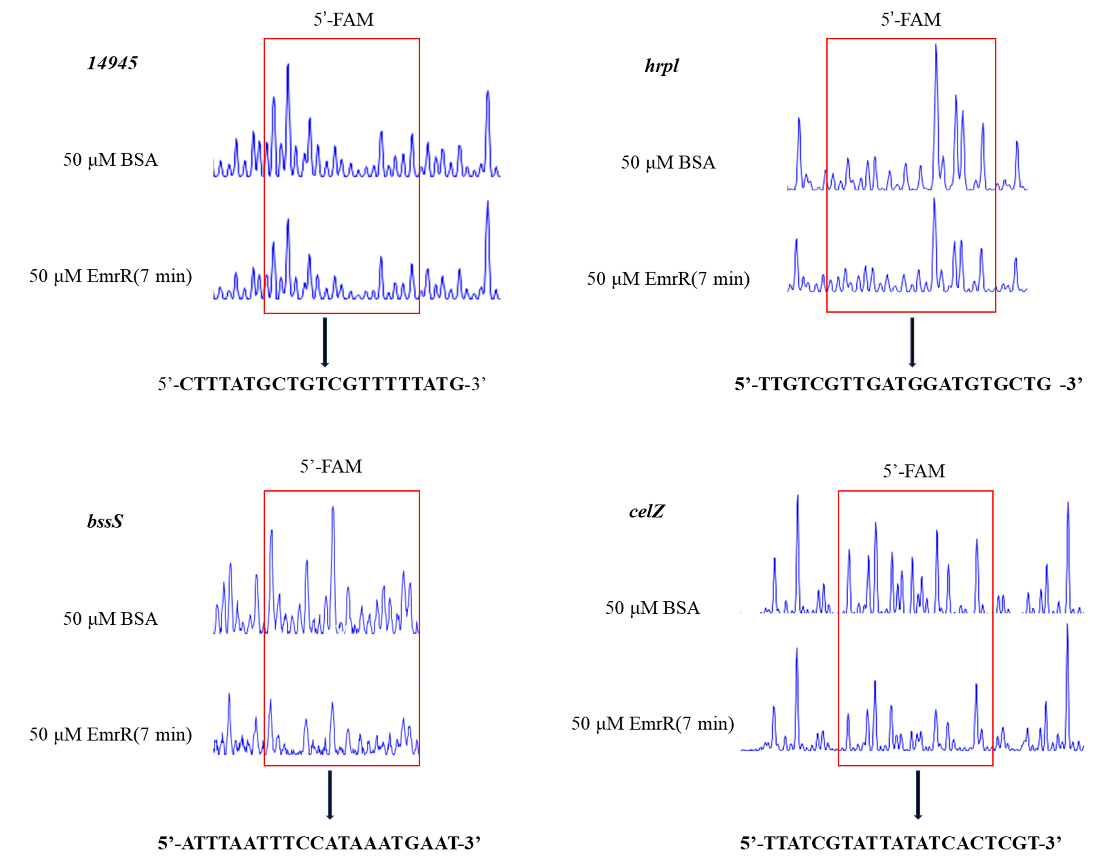


Figure S8. DNase I footprinting analysis. DNase I footprinting analysis was performed using EmrR and the promoter region of *14045*, *hrpL*, *bssS* and *celZ* labelled with 5’-FAM.
