# Supplementary material for: The MarR Family Transcriptional Regulator EmrR Negatively Regulates the Type III Secretion System (T3SS) and Positively Modulates Pathogenicity in Dickeya oryzae
Source: Mol Plant Pathol. 2026 Apr 6;27(4):e70255. doi: 10.1111/mpp.70255 (PMC13053672; doi:10.1111/mpp.70255)
Supplement: Supplementary file 9 — Table S1: Primers used in this study. [file MPP-27-e70255-s004.docx]

**Table S1.** Primers used in this study

| Primers | Sequence (5'-3') | Purpose |
| --- | --- | --- |
| Mutant construction primers | | |
| *emrR*-F | CCTGGGGTTACTGGTATCGTCT | Identification of *emrR* mutant |
| *emrR* -R | CTTCACGGCGGTTCAAGTCACT |  |
| *emrR* -1 | cg**ggatcc**GCATCACTGTACCAACCAGCAC | Amplification of *emrR* upstream sequence |
| *emrR* -2 | TAGAGACGGGGGTTAGAAACCGGGAGCGAACGAACTTTCCA |  |
| *emrR* -3 | TGGAAAGTTCGTTCGCTCCCGGTTTCTAACCCCCGTCTCTA | Amplification of *emrR* downstream sequence |
| *emrR* -4 | gg**actagt**GACGGACGTTGATCATCTGCTG |  |
| pLME-*emrR*-F | ttaggcaccccaggc**ggatcc**GAGACACCATGAATACATCAGTATTATTG | Reporter vectors construction of *emrR* promoter |
| pLME-*emrR*-R | ggaaacagtggacat**ggatcc**GTCTTCGATTGGAGCGAACG |  |
| pLME-*emrAB*-F | ttaggcaccccaggc**ggatcc**CTTCGCGTACCAATGCCACC | Reporter vectors construction of *emrAB* promoter |
| pLME-*emrAB*-R | ggaaacagtggacat**ggatcc**GGGTTTTGATGGCACGGTAG |  |
| pLAFR-*emrR*-F | attacgaattcccgg**ggatcc**GCCTCCATTTGCGCCCTG | Amplification of *emrR* complementary sequence |
| pLAFR-*emrR*-R | acgacggccagtgcc**aagctt**TGGCCTTTGATTGCCGTTC |  |
| pLAFR3-F | tgtgagttagctcactcattaggca | Sequencing primers of pLAFR3 |
| pLAFR3-R | gcgggcctcttcgctattac |  |
| pKNG-F | GACACTGAATACGGGGCAAC | Sequencing primers of pKNG 101 |
| pKNG-R | CCCCTGGATTTCACTGATGA |  |
| *hrpN*-F | GCGTTTATGGCCGCGATTAC | Identification of *hrpN* mutant |
| *hrpN*-R | GTACGGGTCATCCGTTCCG |  |
| *hrpN-*1 | cccctgcaggtcgac**ggatcc**GCAATCCGGTATCGAGCTGG | Amplification of *hrpN* upstream sequence |
| *hrpN-*2 | atcaggcgttcGCTTTGATCGTAATTTGCATAATCTC |  |
| *hrpN-*3 | gatcaaagcGAACGCCTGATAACGTGCCA | Amplification of *hrpN* downstream |
| *hrpN-*4 | cggactatagactat**actagt**CGCTGGATATACCAGTTGCTGA |  |
| *hrpS-*F | CGTGTTTGCGGATTGTCAGG | Identification of *hrpS* mutant |
| *hrpS-*R | CTCGGCGCATTGCCATTAAC |  |
| *hrpS-*1 | cccctgcaggtcgac**ggatcc**CCCATAAATCACGATTAATGGAGA | Amplification of *hrpS* upstream sequence |
| *hrpS-*2 | acaattcgcgGACTACTCCTGTATTTTTCCTGTATTCC |  |
| *hrpS-*3 | aggagtagtcCGCGAATTGTAGCGCGAA | Amplification of *hrpS* downstream |
| *hrpS-*4 | cggactatagactat**actagt**CTAAATAATTCGAGTTGCAGGACAA |  |
| *hrpL-*F | GTCGGGTGTTTCGGGTTTTC | Identification of *hrpL* mutant |
| *hrpL-*R | TAAATCCGTCATCCTGCGGC |  |
| *hrpL-*1 | cccctgcaggtcgac**ggatcc**CTGCTCGCCAAAACTGGCC | Amplification of *hrpL* upstream sequence |
| *hrpL-*2 | ggctgggcTTCATTTACTCTCCATCGTTGTGC |  |
| *hrpL-*3 | agagtaaatgaaGCCCAGCCCCTGCGTGGT | Amplification of *hrpL* downstream |
| *hrpL-*4 | cggactatagactat**actagt**CGCGTAATCAGATCCGGTGA |  |
| *hrpA-*F | CACGGAGATTATCCTGCCGC | Identification of *hrpA* mutant |
| *hrpA-*R | TCGCTACCACCTTAGCGTTG |  |
| *hrpA-*1 | cccctgcaggtcgac**ggatcc**CAATATCCGGGAACTGAAAGCC | Amplification of *hrpA* upstream sequence |
| *hrpA-*2 | ccaactacccacacaacaGATAGATATCTCCAGTTAGCAATTGATTG |  |
| *hrpA-*3 | tcTGTTGTGTGGGTAGTTGGCTACC | Amplification of *hrpA* downstream |
| *hrpA-*4 | cggactatagactat**actagt**CGCATCCATCACATCTTTCGG |  |
| *β*-galactosidase activity assay | | |
| pLME-*emrR*-F | taggcaccccaggc**ggatcc**GAGACACCATGAATACATCAGTATTATTG | Construction of the *emrR* reporter plasmid |
| pLME-*emrR*-R | ggaaacagtggacat**ggatcc**GTCTTCGATTGGAGCGAACG |  |
| pLME-*emrAB*-F | ttaggcaccccaggc**ggatcc**CTTCGCGTACCAATGCCACC | Construction of the *emrAB* reporter plasmid |
| pLME-*emrAB*-R | ggaaacagtggacat**ggatcc**GGGTTTTGATGGCACGGTAG |  |
| pLME-vc2-F | ttaggcaccccaggc**ggatcc**ATAACGCCTATATTTGAAAGCTTGTC | Construction of the vc2 reporter plasmid |
| pLME-vc2-R | ggaaacagtggacat**ggatcc**TTTAATACTGGTTTATCCATGCTGTTAG |  |
| pLME-*hrpA*-F | ttaggcaccccaggc**ggatcc**CCGGGAACTGAAAGCCGC | Construction of the *hrpA* reporter plasmid |
| pLME-*hrpA*-F | ggaaacagtggacat**ggatcc**AGCGGTAGCGTCCAGGTATTT |  |
| pLME-*hrpN*-F | ttaggcaccccaggc**ggatcc**GCGCGCTGGTCGCTGCAA | Construction of the *hrpN* reporter plasmid |
| pLME-*hrpN*-F | ggaaacagtggacat**ggatcc**CCCCAGTCCCGGCCCGGC |  |
| pLME-*hrpL*-F | ttaggcaccccaggc**ggatcc**GCCAAAACTGGCCGTGACT | Construction of the *hrpL* reporter plasmid |
| pLME-*hrpL*-F | ggaaacagtggacat**ggatcc**GGTATGAACCTGACACACATCCAA |  |
| EmrR protein expression primers | | |
| pET30a-*emrR*-BamHI-F | gccatggctgatatc**ggatcc**ATGGAAAGTTCGTTCGCTCCA | Prokaryotic expression of EmrR |
| pET30a-*emrR*-HindIII-R | ctcgagtgcggccgc**aagctt**TTAGAAACCGTCCTGCTCCATG |  |
| pET30a -F | TAATACGACTCACTATAGGG | Sequencing primers of pET30a |
| pET30a -R | GCTAGTTATTGCTCAGCGG |  |
| RT-qPCR primers | | |
| 16s1369F | CGGTGAATACGTTCYCGG | Reference gene of qRT-PCR |
| 16s1541R | AAGGAGGTGATCCRGCCGCA |  |
| *zmsA*-F | CAGGATTATCAGTCAGTAGA | qRT-PCR of *zmsA* |
| *zmsA*-R | GTGCTCATTGCTATTCAG |  |
| *zmsK*-F | AGAGAATAACGCTACCAT | qRT-PCR of *zmsK* |
| *zmsK*-R | CTTCAACAACTCATCCAT |  |
| *hrpN-*F | TAAGGAAGATCGTGGACTG | qRT-PCR of *hrpN* |
| *hrpN-*R | TTTCTGATACTGCGGTTTAC |  |
| *hrpS-*F | CGAGCAGTCACTTCATTG | qRT-PCR of *hrpS* |
| *hrpS-*R | GCAATAGTGTGGATGAGTG |  |
| *hrpL-*F | CAGCCGTATTACCGAGAG | qRT-PCR of *hrpL* |
| *hrpL-*R | CAGCAGCATCACTAACATC |  |
| *hrpA-*F | AAATACCTGGACGCTACC | qRT-PCR of *hrpA* |
| *hrpA-*R | TCCGTGATGGATGACATAG |  |
| *hrcC-*F | TATTCTGACTCCGCATCTG | qRT-PCR of *hrcC* |
| *hrcC-*R | AACTGACGACGGTTATCC |  |
| *hrpX*-F | CGCTCAAACCTGTAAACC | qRT-PCR of *hrpX* |
| *hrpX*-R | GGCTGTCTATCGTCACTT |  |
| *hrpJ-*F | GCTATCTCCCTGTTCAGT | qRT-PCR of *hrpJ* |
| *hrpJ-*R | GGACAATACGCTGGTAGTA |  |
| *hrpP-*F | GACATGCCATCGTTACAC | qRT-PCR of *hrpP* |
| *hrpP-*R | CGTCTACGCTACCCAATT |  |
| *emrA-*F | CCCTACTGATGATCCTGTT | qRT-PCR of *emrA* |
| *emrA-*R | TGCCGTAATACCAGATACC |  |
| *emrB-*F | TGCGGAACTTCACTATCG | qRT-PCR of *emrB* |
| *emrB-*R | CCTGTAATAACTGCGGTAAC |  |
| *fliS*-F | CAGGCAATTATGGAAGTGG | qRT-PCR of *fliS* |
| *fliS*-R | CCGTCGTTGGTTGATAAC |  |
| *fliT*-F | CAGCCAGATTACAGTTCCT | qRT-PCR of *fliT* |
| *fliT*-R | GTGCTTCCGTATCCAGTA |  |
| *cheR-*F | ACCGTATTCTCTGGCTATG | qRT-PCR of *cheR* |
| *cheR-*R | TCACTGGCTGTTATCTGG |  |
| *motA*-F | CGTCAGAAGAACTCAGAGA | qRT-PCR of *motA* |
| *motA*-R | CATTCAGGCTGGATAATAGG |  |
| *celZ*-F | AAGTGTTGAACCATTGTC | qRT-PCR of *celZ* |
| *celZ*-R | GTCTCTGCCGTATAGAAT |  |
| bssS-F | TACCGTTGACAGTTATGA | qRT-PCR of *bssS* |
| bssS-R | GCCTCAAGGATAGAGATAA |  |
| *flgM*-F | AAAGTTAAAGCGTAAAGAGGAA | qRT-PCR of *flgM* |
| *flgM*-R | TTAGACTGAGCGTCACTC |  |
| *14945*-F | TATGTCGCTGATAAGATTC | qRT-PCR of *14945* |
| *14945*-R | CGTTAATGGCTAATGTCA |  |
| *14950*-F | TTCATTATGCTGCTACAA | qRT-PCR of *14950* |
| *14950*-R | CTATCTCCCACTCTTCTT |  |
| *10355*-F | ATGACGAGAATATGATGAAG | qRT-PCR of *10355* |
| *10355*-R | ATACTGAGTGACGCATAG |  |
| RT-PCR | | |
| *emrRA*-F | CAGTTGGAAGTGCTGATGCG | RT-PCR of *emrR* and *emrA* |
| *emrRA*-R | GAGCCAGCGTAATCTGTTGC |  |
| *emrAB*-F | TGATGGTTACGTGTCTCGCC | RT-PCR of *emrA* and *emrB* |
| *emrAB*-R | TTATCACTGATCCAGCCGCC |  |
| DNA sequence amplification of promoters | | |
|  |  | Amplification of *emrR* promoter region sequence |
| EmrR-F | TGCAGTACCCGGAAAGACAG |  |
| EmrR-R | GGAGCGAACGAACTTTCCATAGA |  |
| 14945-F | ATCGGTATAATTGATTTGCTTACGA | Amplification of *14945* promoter region sequence |
| 14945-R | AATAACACTCTCCTCAGGCC |  |
| CelZ-F | TCACGCTAATCTCTACAGGTG | Amplification of *celZ* promoter region sequence |
| CelZ-R | GAATGAATCTCCATTTCAGATGG |  |
| bssS-F | TTATGCCGCCTCGAATAACC | Amplification of *bssS* promoter region sequence |
| bssS -R | CACAAGCGGGTGCGTTTG |  |
| HrpL-F | TTTTGCGTTCAACCTGCTCG | Amplification of *hrpL* promoter region sequence |
| HrpL-R | CCATTTCATTTACTCTCCATCGTTG |  |
| 10355-F | CGGTGATTGGTTGGCTATGG | Amplification of *10355* promoter region sequence |
| 10355-R | GGGGCTCGACTCATATTGTTTTTTTTG |  |
| * The underlines are the site of restriction endonuclease | |  |
